# Light-Activated Metal Oxide Gas Sensors: A Review

**DOI:** 10.3390/mi8110333

**Published:** 2017-11-18

**Authors:** Fang Xu, Ho-Pui HO

**Affiliations:** 1 Department of Electronic Engineering, The Chinese University of Hong Kong, Shatin, New Territories, Hong Kong, China; xufangabroad@gmail.com; 2 Department of Biomedical Engineering, The Chinese University of Hong Kong, Shatin, New Territories, Hong Kong, China

**Keywords:** gas sensors, metal oxides, heterostructures, doping, light activation, one-dimensional nanostructures, porous nanostructure, localized surface plasmon resonance

## Abstract

Conductometric gas sensors facilitated by photons have been investigated for decades. Light illumination may enhance device attributes including operational temperature, sensing sensitivity and selectivity. This paper aims to provide an overview on the progress of light-activated gas sensors, with a specific focus on sensors based on metal oxides. The material systems that have been studied include pure metal oxides, heterostructures of semiconductor-metal oxides and metal-metal oxides, and metal oxides with dopant. Other reported works on the use of different nanostructures such as one-dimensional and porous nanostructures, study of sensing mechanisms and the interplay between various factors are also summarized. Possible directions for further improvement of sensing properties, through optimizing the size of nanomaterials, film thickness, light intensity and wavelength are discussed. Finally, we point out that the main challenge faced by light-activated gas sensors is their low optical response, and we have analyzed the feasibility of using localized surface plasmon resonance to solve this drawback. This article should offer readers some key and instructive insights into the current and future development of light-activated gas sensors.

## 1. Introduction

Gas sensors play an important role in various disciplines ranging from personal safety, medical diagnosis, environmental monitoring to industrial process control [1,2,3,4,5,6]. Compared to other types of gas detection techniques which typically make use shifts in optical and electrical capacitance properties [7,8], the conductometric gas sensor attracts much attention due to several advantages such as simplicity in measurement setup and the ease of miniaturization for portable instruments. The conductometric gas sensor typically consists of a sensor head and a resistance measurement system. The sensor head is composed of a sensing element, electrodes and a heater. The material for the sensing element is usually a metal oxide semiconductor. Resistance of the sensing element changes upon exposing it to one or a class of vapor species. For *n*-type sensing materials like ZnO and SnO_2_, the resistance decreases upon exposure to reductive gases such as CO, H_2_, ethanol vapor and increases upon exposure to oxidizing gas like O_2_, NO_2_, O_3_. The sensing results are opposite for *p*-type sensing materials. The relationship between resistance of the sensing material and the gas type is illustrated in Table 1. The most reported metal oxide semiconductors that are being used as sensing element materials include: SnO_2_, ZnO, TiO_2_, In_2_O_3_, WO_3_, CuO, Fe_2_O_3_, MoO_3_, while the most reported target gases are H_2_, CO, NH_3_, H_2_S, humidity, O_2_, NO_2_, NO_X_, and vapors of ethanol, formaldehyde, acetone, methanol.

Five parameters are important for a conductometric gas sensor: response, response time, recovery time, selectivity and working temperature. There are several different definitions of the sensing response: change of conductance [9], change of current [10,11,12,13,14], and change of the resistance, which is mostly used in practical sensors. Based on the shift of resistance, the response of *n*-type material when exposed to a reductive gas is defined as R_a_/R_g_ [15] or (R_a_ − R_g_)/R_g_ [16], where R_a_ and R_g_ are the resistances of the device in air and in the target gas atmosphere respectively. For the gas sensors based on *p*-type materials, the response due to a reductive gas is defined as R_g_/R_a_ [17] or (R_g_ − R_a_)/R_a_. For detecting oxidizing gases, the definitions of response are interchanged between *n*-type materials and *p*-type materials. Different definitions of response are illustrated in Table 2. Generally, the response time (T_res_) and recovery time (T_rec_) are defined as time spent by a sensor to achieve 90% of the total resistance change during the adsorption and desorption process respectively [15,17,18]. In some cases, the work defines the response time and recovery time as the time required for the sensor to achieve 90% of the total current change [19] or 90% of the total response change [20].

An optimal conductometric gas sensor should meet the requirements of large sensing response, low working temperature and high selectivity to the target analytes. The mechanism for the resistance change of a conductometric gas sensor upon exposure to a gas is due to chemisorption and reaction of gas molecules on surface of the sensing element. Take ZnO, an *n*-type semiconductor material, as an example, when the material is exposed to air, oxygen in air will be adsorbed on the surface of ZnO and capture electrons from the conduction band of ZnO to form oxygen ion species. Activities of different kinds of oxygen ions are dependent on the working temperature. It is reported that O_2_^−^ is formed at temperature lower than 150 °C and O^−^, O^2−^ are formed at higher temperature [21,22,23]. A depletion layer is formed in ZnO, which causes the resistance to increase. Reactions between the oxygen ions and the target gas molecules ultimately lead to the sensor’s signal output. A reducing gas will be oxidized by oxygen ions and released electrons back to the conduction band of ZnO, thus decreases the resistance. An oxidizing gas will be reduced by capturing electrons, thus increasing the sensor’s resistance. After completing the sensing response process, the sensing element can be regenerated by exposing it to air again, as oxygen will be adsorbed on the surface to form oxygen ions again. In the light of this reaction step, the sensing response is highly dependent on several key parameters including working temperature, surface-to-volume ratio, specific surface area, surface active sites, size of the nanostructure and concentration of energetic carriers [24]. The reported schemes for improving the performance of conductometric gas sensors include the use of different nanostructures [25,26,27], controlling the size of nanoparticles [28], combining different materials by synthesizing heterostructures [29] and by doping.

Consequent to the generation of oxygen ions, which governs the operation of conventional conductometric gas sensors based on metal oxide semiconductors, one typically needs to heat the sensing element to a working temperature of at least 150 °C [2,3,6]. The thermal activation of gas sensor causes several disadvantages [24]. A heating element should be added to the gas sensor head, which increases device complexity. High working temperatures also sacrifices device lifetime and long-term stability of sensing performance as this will result in regrowth of nanomaterials. It will also limit the sensor’s application in the detection of flammable or explosive analytes because of safety issues. Thus, an important research aim in the conductometric gas sensor is to lower the working temperature [24,30,31,32,33,34], Photon activation hence becomes an obvious choice.

This review aims to provide a summary on recent development of light-activated conductometric gas sensors that are based on metal oxide semiconductors. Since the sensing response of photo-activated gas sensors is generally quite low compared to that of conventional thermal energy activated sensors, several approaches have been reported to improve the situation. Here, we have reviewed various nanomaterials or nanostructures, including pure metal oxides, heterostructures made of different materials and metal oxides with doping. For the case of pure metal oxides, sensors made of 1-dimensional and porous nanostructures are discussed. The sensing properties of these materials are compared and the mechanisms of the performance improvement are discussed. Finally, an outlook on the potential development of plasmon-assisted conductometric gas sensors is presented.

## 2. Pure Metal Oxide Materials for Gas Sensing

### 2.1. Pure Metal Oxide Nanoparticles

Metal oxides for gas detection was first found in 1960s by Tetsuro Seiyama [35]. The researcher used ZnO thin films for the detection of several gases including toluene, benzene, CO_2_ at elevated temperature higher than 400 °C. After that, numerous research efforts have been done in the field of metal oxides for conductometric gas sensors. In recent years, increasing attention has been given to the development light-activated conductometric gas sensors for lowering the working temperature. Here, we have summarized light-activated gas sensors based on pure metal oxides of different kinds of materials in the light of their level of their popularity.

#### 2.1.1. ZnO

Despite that SnO_2_ is the most popular material for thermal energy activated gas sensors, which has already been used for research, theoretical study and commercial device, ZnO drew attracted more attention in the field of light-activated gas sensors.

**Figure 1:**
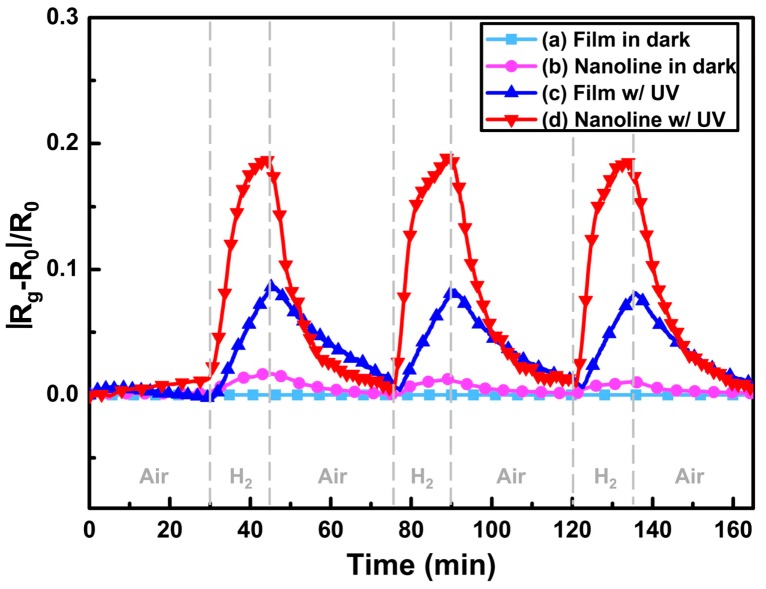
100 ppm H_2_ sensing response plot of (**a**) ZnO thin film in dark; (**b**) 400 nm wide ZnO line in dark; (**c**) ZnO thin film under ultraviolet (UV) light; and (**d**) 400 nm wide ZnO line under UV light. Reproduced with permission from [36].

**Figure 2:**
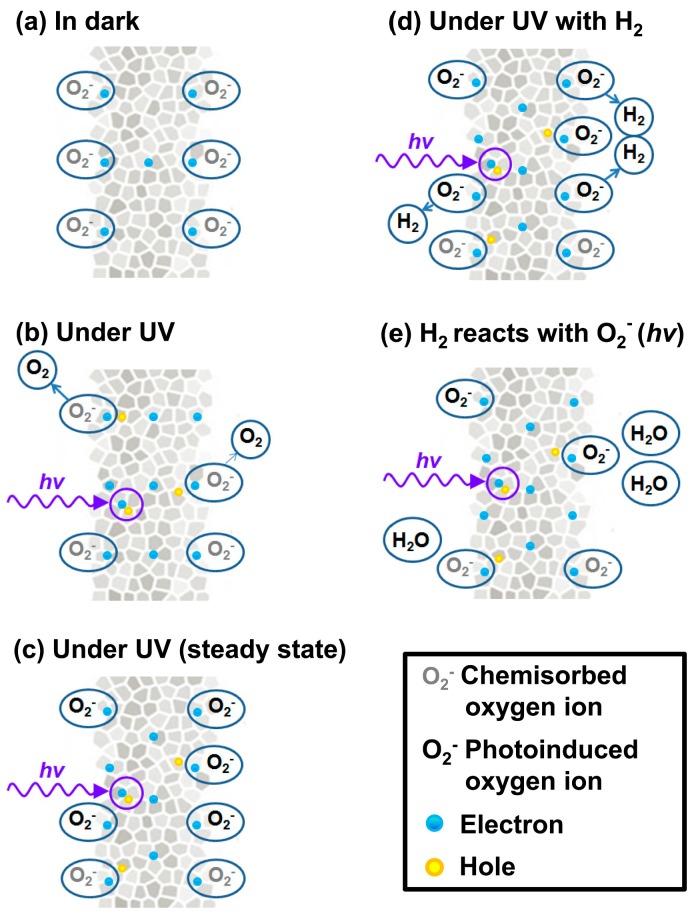
Diagram of the UV-activated room-temperature gas sensing mechanism.(**a**) In dark; (**b**) Under UV; (**c**) Under UV (steady state); (**d**) Under UV with H_2_; (**e**) H_2_ reacts with O_2_^−^ (*hv*). Reproduced with permission from [36].

**Figure 3:**
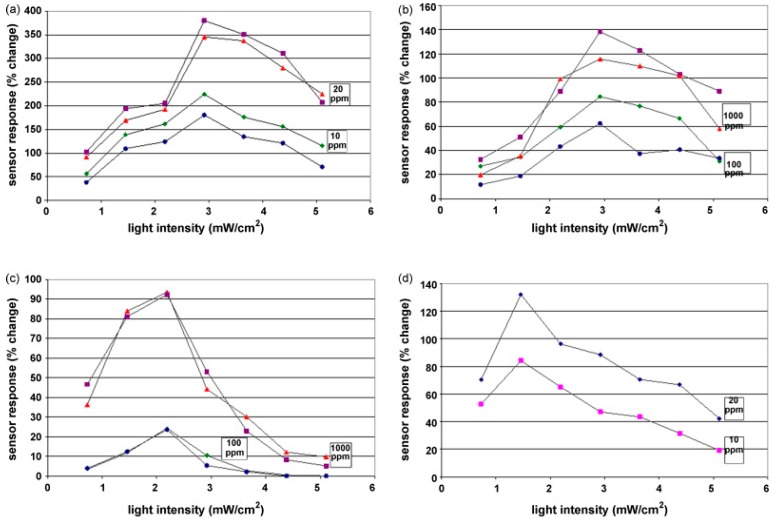
Sensing response (% change in the baseline current) vs. light intensity . Two different sensors based on ZnO were exposed to (**a**) hexane; (**b**) propane; (**c**) methane; (**d**) ethanol vapors of different concentrations. Reproduced with permission from [38].

**Figure 4:**
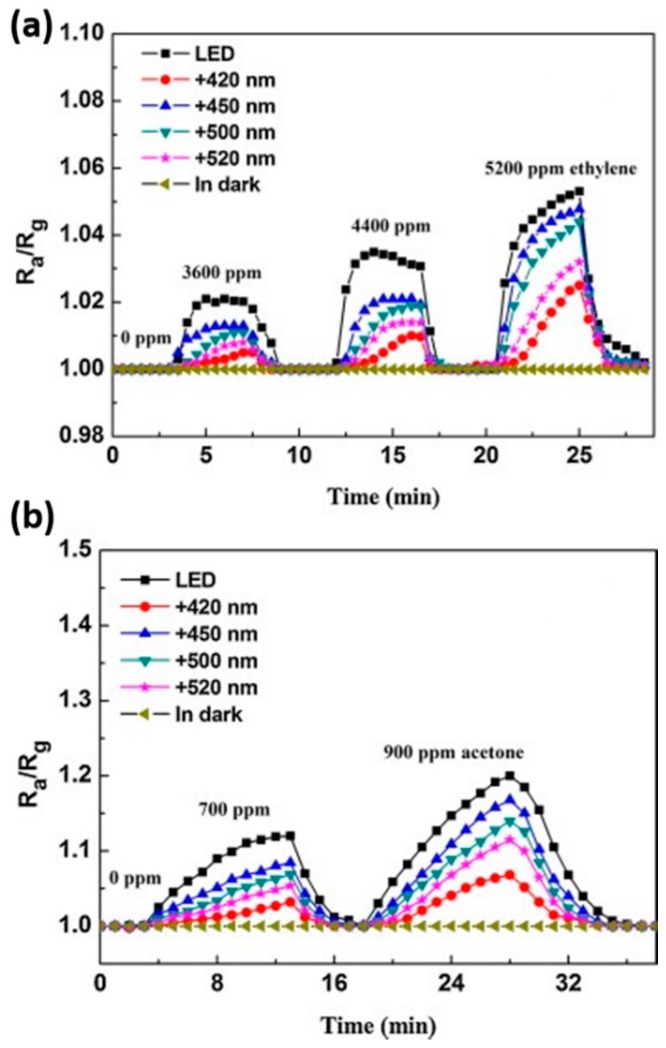
Sensing response of ZnO sensor to different concentrations of (**a**) ethylene and (**b**) acetone in air atmosphere in dark or under visible-light irradiation of different wavelengths. Reproduced with permission from [37].

Fan compared the H_2_ sensing performance of ZnO at room temperature with and without ultraviolet (UV) irradiation, and it was reported that UV light improved the sensing response and recovery rate [36]. As shown in Figure 1, ZnO thin film showed no obvious response to H_2_ in dark and sensing was immediately activated under UV irradiation. They attributed the sensing improvement to photo-induced oxygen ions: O_2_^−^(hν) and the proposed sensing mechanism is shown in Figure 2. O_2_ molecules are chemisorbed on the surface of ZnO and they capture electrons from ZnO to form oxygen ions (O_2_^−^) in dark (Figure 2a): O_2_ + e^−^ → O_2_^−^. With UV illumination, electron-hole pairs are generated and the holes react with the pre-chemisorbed O_2_^−^ to form oxygen molecules which will be desorbed from the surface of ZnO: h^+^ + O_2_^−^ → O_2_. At the same time, new oxygen molecules will be adsorbed and capture the photoelectrons to form photo-induced oxygen ions (O_2_^−^(hν)): O_2_ + e^−^(hν) → O_2_^−^(hν) (Figure 2b). After reaching equilibrium of oxygen adsorption and desorption, the resistance of ZnO reaches a steady state (Figure 2c). O_2_^−^(hν) ions are highly reactive and loosely bound to the ZnO surface, which can be reduced by H_2_ molecules to release electrons back to ZnO (Figure 2e): 2H_2_ + O_2_^−^(hν) → 2H_2_O + e^−^. Resistance of ZnO hence decreases under this reaction. After the injection of air, oxygen molecules are adsorbed on the surface of ZnO and capture photo-induced electrons, which will cause resistance to increase again. This is the recovery process of the conductometric gas sensor. This operation mechanism is highly accepted to explain the photo-activated conductometric gas sensor [36,37]. The sensing performance of ZnO nanolines is higher than that of ZnO thin film in both dark and UV irradiation conditions as shown in Figure 1. The better performance is due to its smaller grain size which provide larger surface-to-volume ratio. De Lacy Costello used ZnO nanoparticles to detect various gases like hexane, propane, methane, ethanol, toluene, acetaldehyde, acetone and pentane at room temperature under ultraviolet light-emitting diode (UV-LED) activation, and they reported that the sensing performance did not simply increase with light intensity. In fact, the sensor exhibited maximum response at different light intensity levels depending on the target gas [38]. This conclusion was also reported and analyzed by other groups [38,39,40,41]. In fact, gas-dependent optimal light intensity can be used to tune the sensors’ selectivity (Figure 3) [38]. However, de Lacy Costello did not provide any explanation for this phenomenon. Apart from light intensity, the sensing performance was also found to be wavelength dependent, as this also reported by other researchers [9]. Instead of using UV illumination, Geng reported sensing of acetone and ethylene at room temperature with visible light activation [37]. Their ZnO sensors exhibited no room temperature response to both acetone and ethylene vapors in the dark, even at very high concentration levels. Response to both ethylene and acetone was observed under irradiation of a white LED lamp and visible light with different wavelengths ranging from 420 nm to 520 nm (Figure 4). Since the energy of photons from visible light is smaller than the bandgap of ZnO, it was not possible to explain the visible light activation with intrinsic absorption. The photo response to visible light was therefore attributed to the non-intrinsic absorption due to native defects in ZnO. Electrons in valence band of ZnO could absorb energy from two or more electrons and be excited to the conduction band. These photo-electrons facilitated the gas sensing at room temperature.

**Figure 5:**
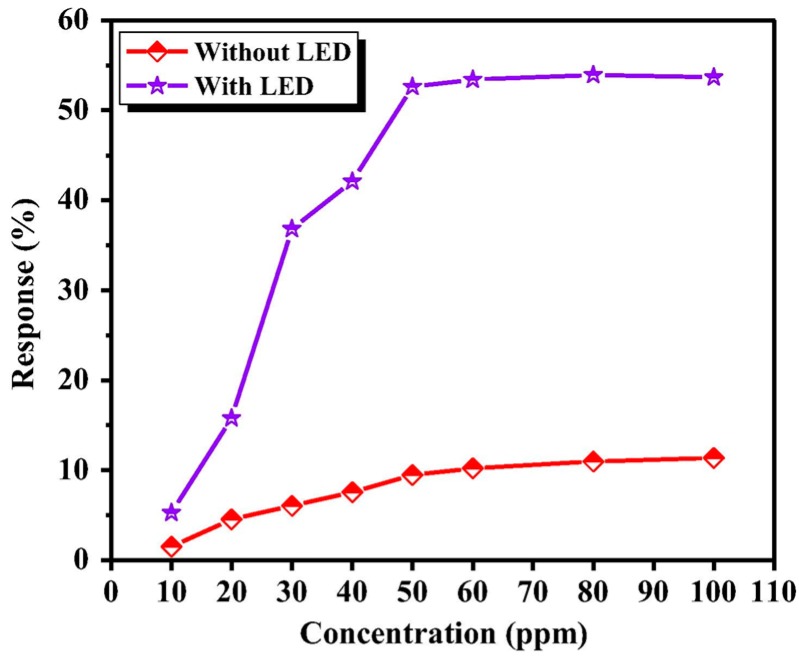
Response of ZnO with and without LED irradiation towards different concentration levels of hydrogen peroxide. Reproduced with permission from [42].

**Figure 6:**
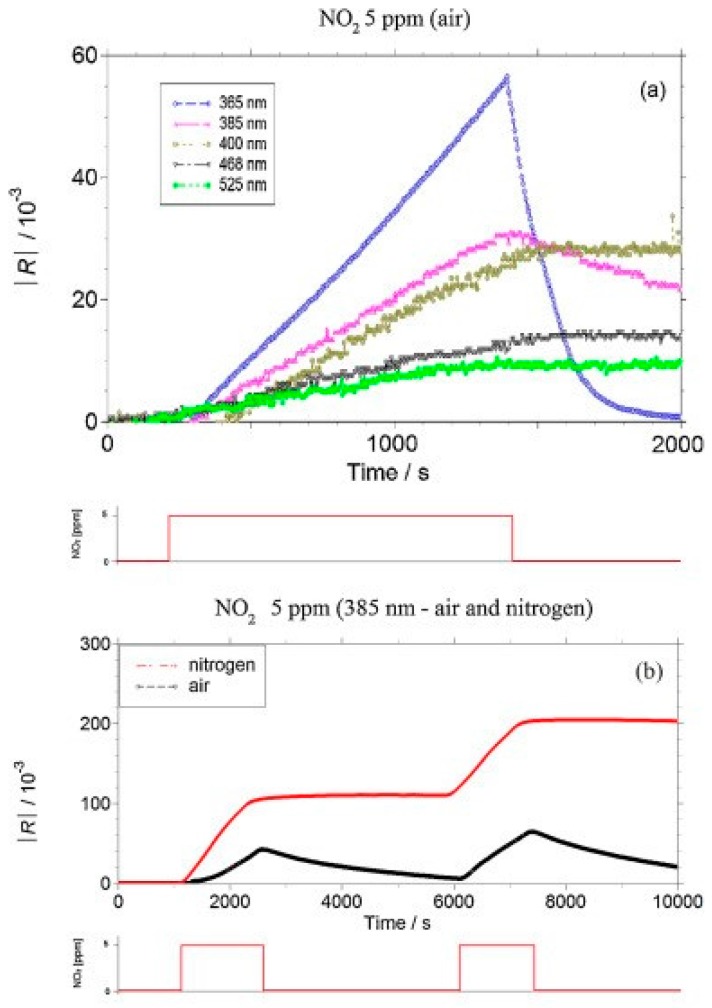
5 ppm NO_2_ sensing plot of ZnO thin film at room temperature as a function of (**a**) light wavelength; (**b**) background gas. Reproduced with permission from [9].

**Figure 7:**
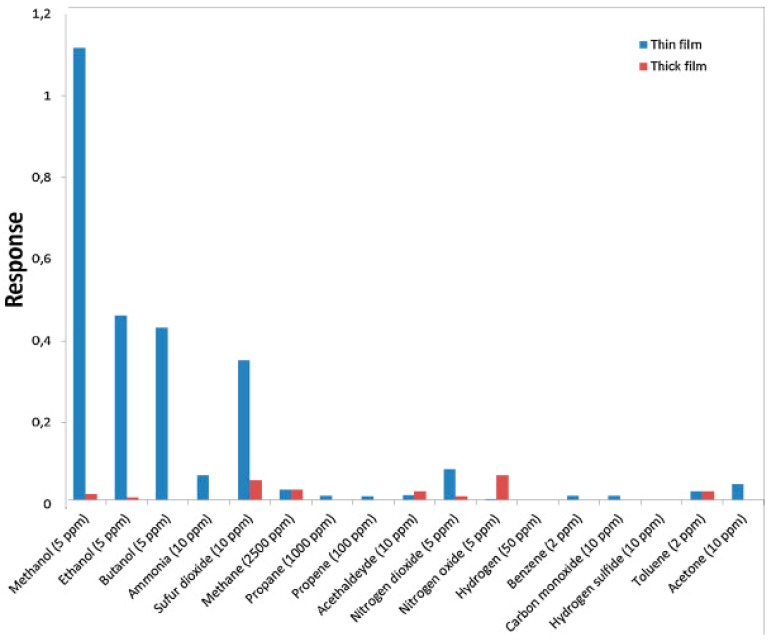
Comparison of sensing response towards various gas samples for the cases of thin and thick ZnO film. Reproduced with permission from [9].

**Figure 8:**
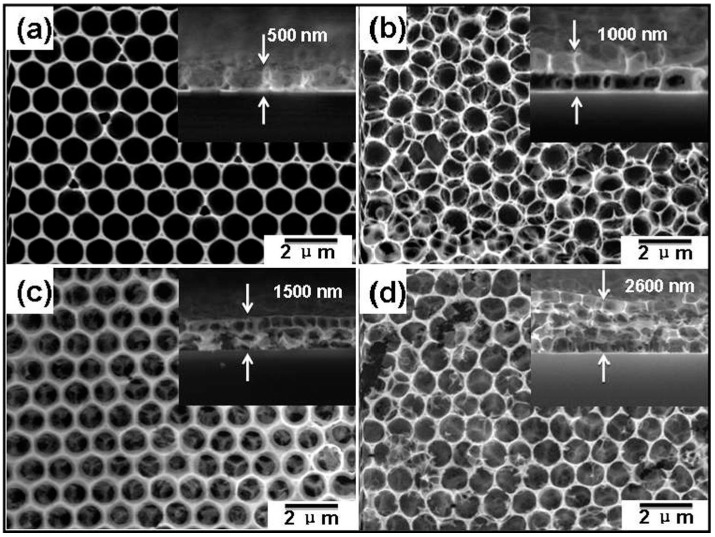
Field emission scanning electron microscopy (FESEM) images of ZnO porous thin film with different thicknesses on silicon wafer substrate, which was prepared by a layer-by-layer strategy based on solution-dipping of monolayer polystyrene (PS) template and transfer. (**a**) Monolayer; (**b**) Bilayer; (**c**) Trilayer; and (**d**) Tetralayer. The insets are the corresponding cross-sectional images. Reproduced with permission from [43].

**Figure 9:**
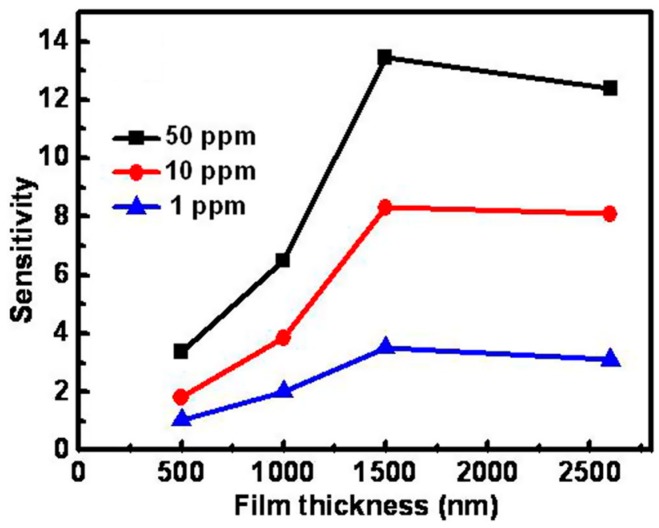
Influence of film thickness on the response of porous ZnO thin film towards NO_2_ at room temperature under UV irradiation. Reproduced with permission from [43].

**Figure 10:**
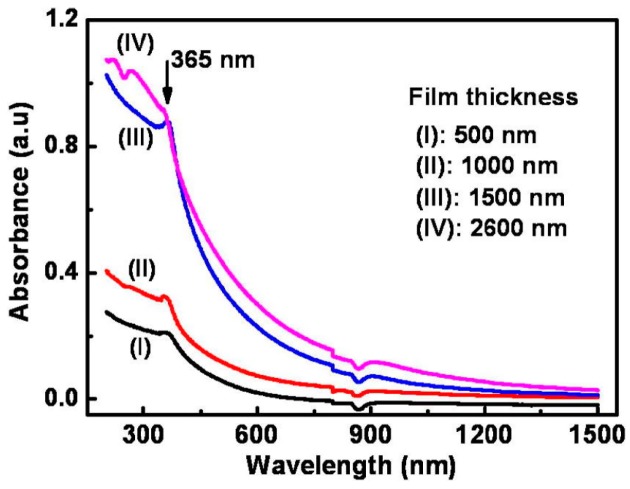
Optical absorbance spectra of porous ZnO porous film with different thicknesses. Reproduced with permission from [43].

Compared to reductive gases, we found more research efforts on studying light-activated sensing of oxidizing gas by ZnO [9,42,43,44,45]. Parthasarathy used ZnO nanospheres to fabricate thin films for detecting H_2_O_2_ at room temperature in conditions with and without light illumination. The white LED enhanced sensing response by increasing carrier concentrations (Figure 5) [42]. Fabbri fabricated a ZnO thin film for NO_2_ sensing at room temperature with light illumination at different wavelengths as shown in Figure 6a, and found that the sensing response was wavelength dependent. Maximum response was activated by photons at 365 nm, which matched the bandgap energy of ZnO. The sensing performance was also influenced by the gas type as shown in Figure 6b. They reported that the NO_2_ sensing response is higher in N_2_ than in the air but irreversible in N_2_, this result was in agreement with the widely accepted sensing mechanism which involves oxygen adsorption and desorption. The sensing response towards various gas samples were compared using thin and thick ZnO films. The authors claimed that the thin film case exhibited higher response because the thick film sample had a layer thickness well beyond the light penetration depth (Figure 7) [9]. This film-thickness dependence was systematically studied by Su et al. through both experiments and theoretical calculation [43]. They fabricated ordered porous ZnO arrays with four different film thicknesses for NO_2_ sensing with UV illumination (Figure 8). The sensing response increased at first and then decreased with increasing film thickness (Figure 9). They theoretically calculated the light penetration depth (h) through analyzing the light intensity decay in the material. The optimal film thickness (H) at which the materials exhibited maximum sensing response should be equal to the light penetration depth. Their result was consistent with the optical absorbance spectra which showed that the absorbance intensity did not increase at 365 nm when the film was thicker than 1500 nm, which was the calculated penetration depth for 365 nm radiation in their ordered porous ZnO samples (Figure 10). The optimal film thickness was dependent on the material, the nanostructure and the wavelength of the incident light.

#### 2.1.2. SnO_2_

**Figure 11:**
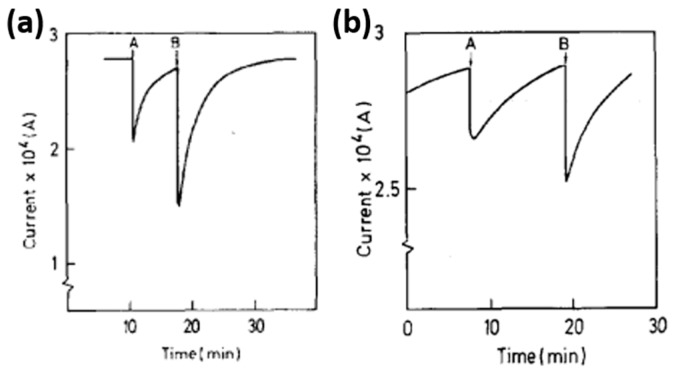
Response of SnO_2_ towards vapors of acetone and trichloroethylene (**a**); trichloroethylene and acetone (**b**) under UV excitation. The short-wavelength cut-off of the excitation spectrum is at approximately 200 nm (**a**) and 230 nm (**b**). Reproduced with permission from [49].

**Figure 12:**
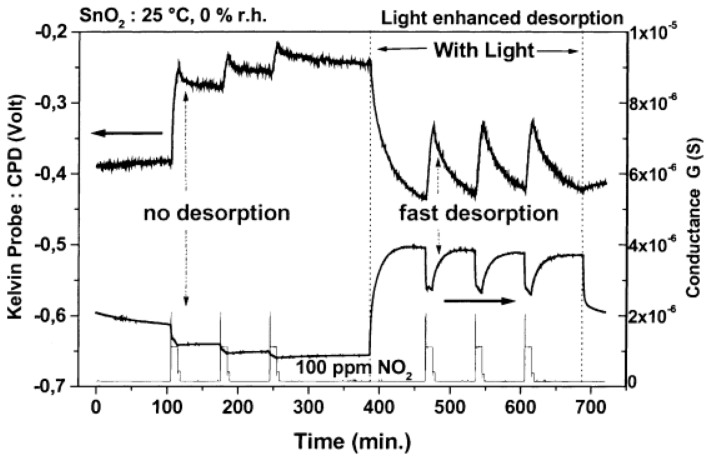
Gas response of SnO_2_ towards 100 ppm NO_2_ with and without illumination at 25 °C and 0% relative humidity. Conductance and contact potential difference (CPD) measurements are shown. Reproduced with permission from [50].

**Figure 13:**
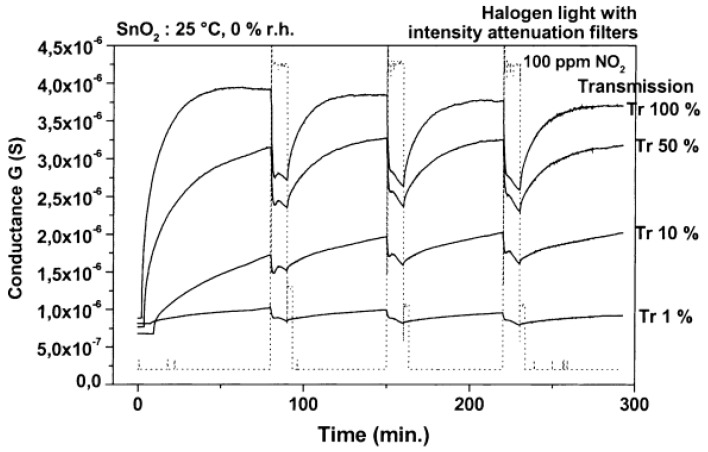
Conductance response of SnO_2_ towards 100 ppm NO_2_ under different light intensities. Reproduced with permission from [50].

**Figure 14:**
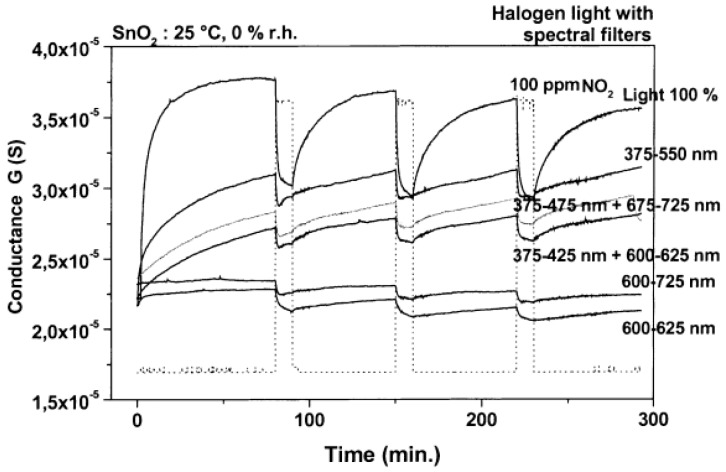
Conductance response of SnO_2_ towards 100 ppm NO_2_ with illumination at different wavelengths. Reproduced with permission from [50].

SnO_2_ is a popular metal oxide for conductometric gas sensor. Light irradiation on SnO_2_ was found to enable room temperature sensing, enhance response, increase sensing rate as well as improve gas selectivity [46,47,48]. Jeng reported UV LED activated O_3_ sensing at room temperature and attributed the sensing mechanism to photo-induced adsorption and desorption as mentioned earlier [48]. Ao reported the Hg lamp irradiation improved the sensing response and sensing speed of SnO_2_ towards H_2_ in both vacuum and in air [47]. Faglia found that monochromatic light improved sensor selectivity through measuring the sensing response towards different gases with and without light illumination [46]. Saura studied gas sensing properties of SnO_2_ pyrolytic films towards acetone and trichloroethylene vapors under UV irradiation at different wavelengths. They claimed a wavelength-controlled selectivity by comparing the sensing response towards the two vapors between different wavelength (Figure 11) [49]. They also reported that the light-activated gas sensing was due to the photodesorption, which was also reported by Anothainart and Comini [16,50]. Anothainart studied NO_2_ sensing performance of SnO_2_ at room temperature with and without halogen lamp illumination by conductance and work function measurements. As shown in Figure 12, there was no desorption of NO_2_ without light illumination and a fast desorption after light illumination. The photon induced desorption is dependent on light intensity and wavelength (Figure 13 and Figure 14). Higher light intensity accelerated the desorption process, which was only activated by photons with wavelength shorter than 600 nm.

#### 2.1.3. TiO_2_

**Figure 15:**
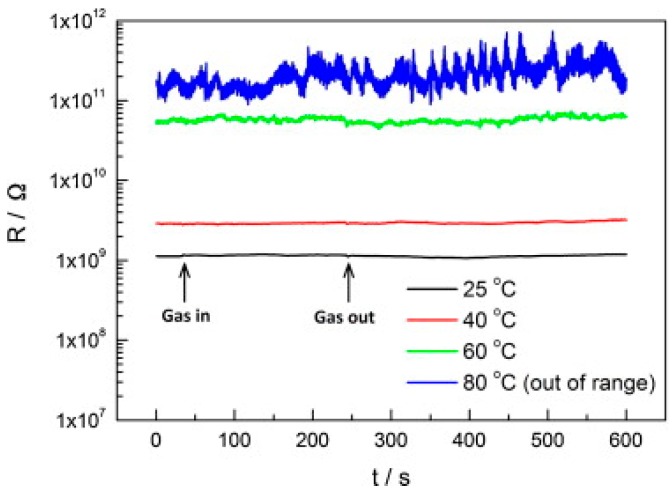
100 ppm formaldehyde sensing results using TiO_2_ with humidity at 13.68 mg/L vs. working temperatures without UV irradiation. Reproduced with permission from [51].

**Figure 16:**
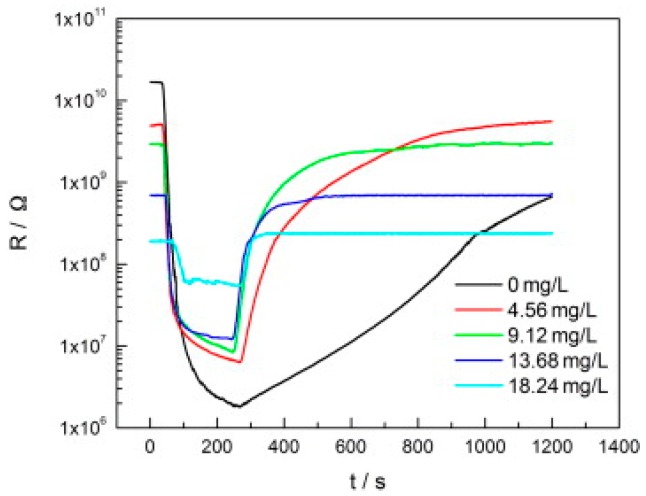
100 ppm formaldehyde UV-activated sensing results for TiO_2_ vs. humidity at room temperature. Reproduced with permission from [51].

**Figure 17:**
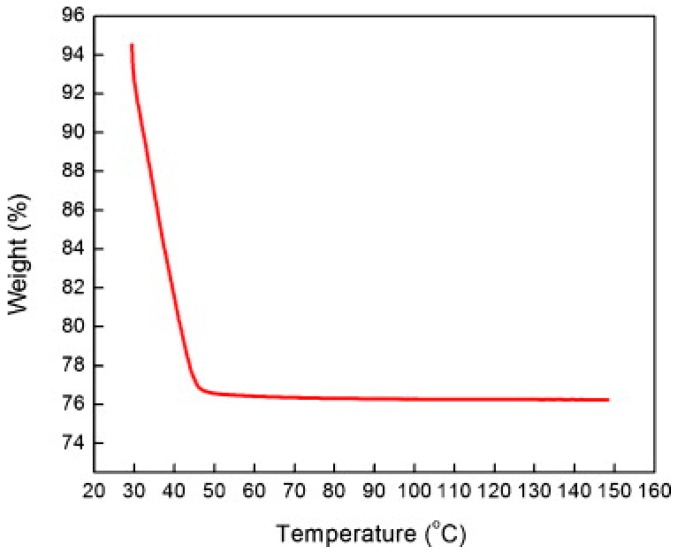
Thermal gravimetric analysis (TGA) results of TiO_2_. Reproduced with permission from [51].

TiO_2_ was studied in light-activated gas sensor. Zhang studied formaldehyde sensing property of TiO_2_ with UV light activation at different temperatures. They found that there was no response without UV irradiation at operation temperature between 25 °C and 80 °C (Figure 15). UV-activated TiO_2_ for sensing of formaldehyde at room temperature was found to be highly dependent on humidity (Figure 16). They studied the reason with thermal gravimetric analysis (TGA) measurement of TiO_2_ and found that the adsorbed water molecules easily physically desorbed from the surface of TiO_2_ at temperatures lower than 45 °C (Figure 17). They modulated the operation temperature ranging from 5 °C to 80 °C and find that 60 °C was the temperature at which sensor performance exhibited lowest humidity dependence [51].

**Figure 18:**
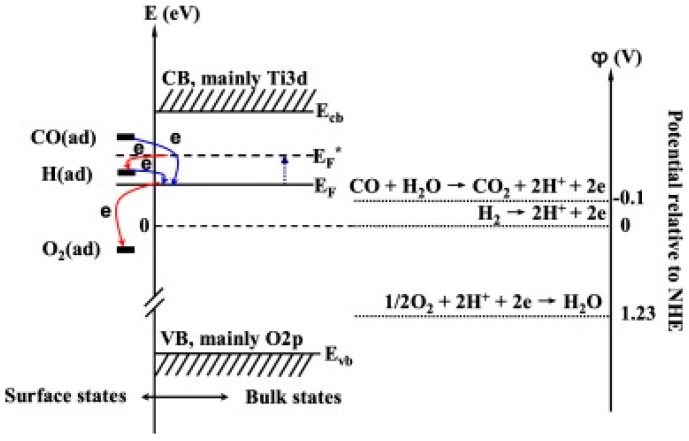
Schematic energy level characterizing the surface and bulk electronic states of TiO_2_, as well as the oxidation potential value of CO and H_2_. The adsorption of H_2_ causes the elevation of Fermi level (from EF to EF* (a quasi-Fermi level)) under UV irradiation, resulting in H_2_ accepting electrons from TiO_2_, while CO still donate electrons to TiO_2_. Reproduced with permission from [52].

It is generally accepted that reductive gases lead to decreasing the resistance of a *n*-type sensing material because reductive gas molecules donate electrons to the sensing material during the reaction. However, Peng studied sensing of TiO_2_ towards CO and H_2_ and found that H_2_ adsorption on TiO_2_ caused the increase of resistance. By analyzing the energy levels of TiO_2_ and the adsorbed gases, they pointed out that photocatalytic oxidation of a reactant was not only dependent on the photo-electrons, the analyte, the sensing materials but also the relationship between the energy of the adsorption state and the fermi energy level of the sensing material. Only when the adsorption energy state was higher than Fermi energy of the material, as the CO had done here, the gas would donate electrons to the sensing material. Here H_2_ molecules showed a lower adsorption energy than the Fermi level of TiO_2_. Thus, it acted as an electron acceptor which caused the resistance of TiO_2_ to increase (Figure 18) [52]. A similar result was observed by Wang, who used vertically aligned ZnO nanowires for CO sensing with light illumination at low intensity. Different from other CO sensing results where CO acts as the reducing gas, here CO caused the resistance of ZnO to increase more after removing the LED source, which indicated that CO worked as an oxidizing gas that trapped electrons from ZnO [53]. In conclusion, it is believed that the reported results confirm that there is no absolute measure on whether an analyte is reductive or oxidative, it is a question of how the energy levels of the analyte are relative to the sensing material.

#### 2.1.4. WO_3_

**Figure 19:**
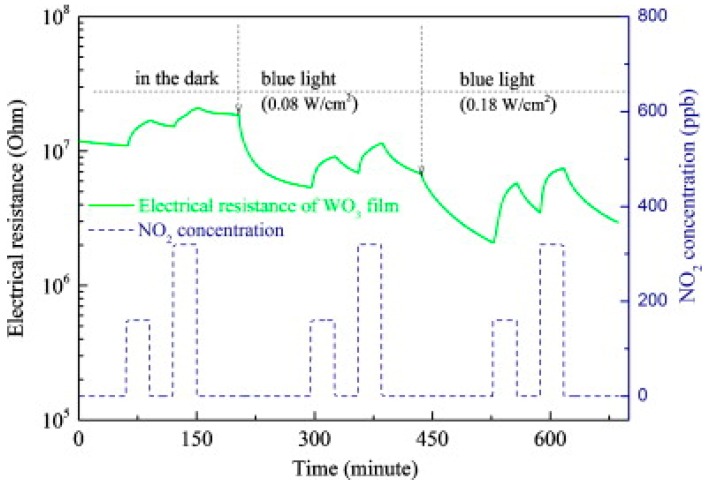
NO_2_ sensing curve vs. blue light intensity. Reproduced with permission from [56].

In recent years, WO_3_ has attracted increasingly attention for gas sensing applications [54,55,56]. Under visible light illumination, WO_3_ exhibited enhanced sensing response and faster sensing ratio to NO_2_ as compared to the case without photons, and the sensing response increased with the light intensity, as reported by Zhang [56] (Figure 19). It was also shown that the sensing was influenced by light wavelength and humidity. The blue light at λ = 480 nm resulted in fasted sensing speed.

#### 2.1.5. In_2_O_3_

**Figure 20:**
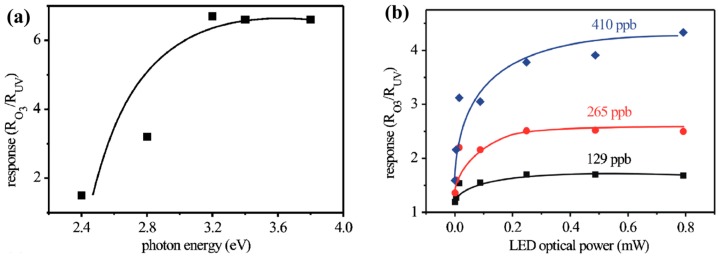
O_3_ sensing response vs. (**a**) photon energy; (**b**) LED light power. Reproduced with permission from [57].

**Figure 21:**
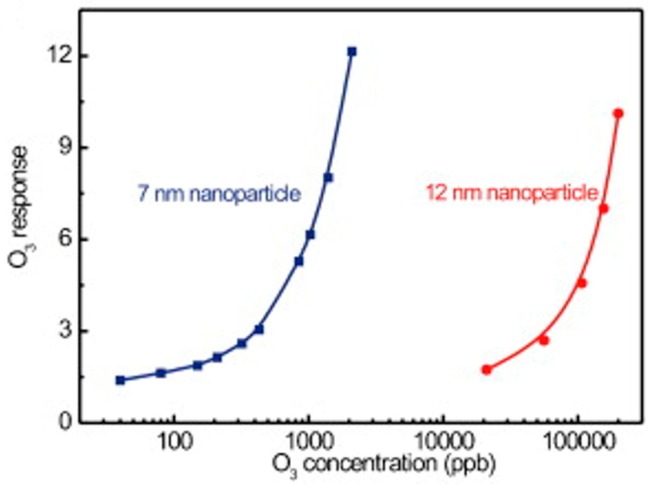
O_3_ response vs. size of In_2_O_3_ nanoparticles. (**blue** line) 7 nm and (**red** line) 12 nm. Reproduced with permission from [58].

In_2_O_3_ was investigated for light-activated gas sensors and the analyte was ozone. Wang fabricated sensors with ultrathin layers of In_2_O_3_ nanoparticles integrated with GaInN/GaN based LED and investigated the ozone response as a function of wavelength and optical power. The response of the sensor increased with increasing both photon energy and light intensity (Figure 20) [57]. They later reported that light-activated In_2_O_3_ sensor exhibited a sensing property dependent on the size of the nanoparticle. They compared the sensing characteristics of 7 nm and 12 nm In_2_O_3_ nanoparticles towards O_3_ at room temperature. 7 nm nanoparticles showed a lower limit of detection at 10 ppb, compared to 1 ppm for 12 nm ones. Moreover, to obtain the same response value, a sensor based on 12 nm In_2_O_3_ nanoparticles required a concentration ~100 times larger than that of 7 nm nanoparticles (Figure 21). This size dependent sensing property was explained by the larger surface area of 7 nm nanoparticles and higher porosity of the sensing layer, thus resulting in more O_3_ molecules being adsorbed on the surface [58].

#### 2.1.6. Summary

In the above section, we have summarized the progress of light-activated gas sensor based on metal oxide nanoparticles, namely ZnO, SnO_2_, TiO_2_, WO_3_ and In_2_O_3_. A detailed summary of these reported work is shown in Table 3. Light illumination may activate gas sensing at room temperature, enhance sensing response, sensing speed and selectivity. Several factors affecting the sensing characteristics were discussed. They include light intensity, wavelength, size of nanoparticles and film thickness. The photoadsorption-desorption model has been widely accepted as the mechanism of light-activated gas sensor.

### 2.2. Metal Oxides with One-Dimensional Nanostructures

One dimensional nanostructures are favored in many applications including conductometric gas sensing. One-dimensional (1D) nanostructures may enhance sensing response, speed of response and speed of recovery and lower limit of detection. Such improvements are associated with high surface-to-volume ratio, high specific surface area, comparable scale between each unit of sensing material and the depletion layer, fast electron transfer path and excellent crystallinity [25]. Many published works reported the use of 1D nanostructured metal oxides in both thermal and light-activated conductometric gas sensors. Here we discussed the 1D nanostructured metal oxides in the development of light-activated gas sensors in the sequence of their relative popularity: nanowires, nanorods, nanofiber, nanotube and nanoribbon.

#### 2.2.1. Nanowires

**Figure 22:**
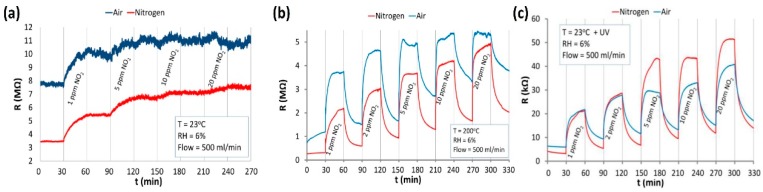
NO_2_ sensing of ZnO nanostructures in different atmospheres of synthetic air and nitrogen at: (**a**) room-temperature (RT); (**b**) elevated temperature of 200 °C; (**c**) RT and UV irradiation. Reproduced with permission from [44].

**Figure 23:**
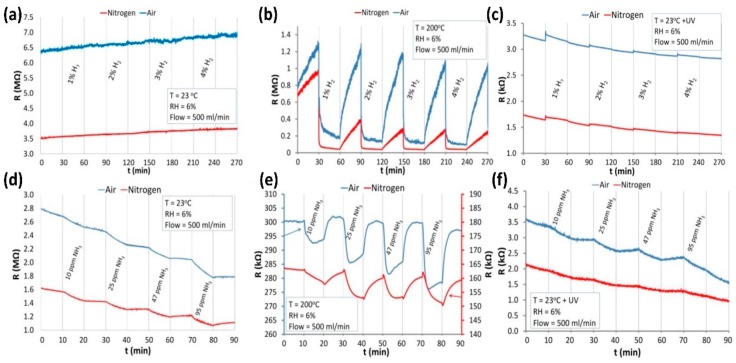
H_2_ sensing of ZnO nanostructures in different atmospheres of synthetic air and nitrogen: (**a**) RT; (**b**) evaluated temperature of 200 °C; (**c**) RT and UV irradiation. NH_3_ sensing of ZnO nanostructures in air and nitrogen: (**d**) RT; (**e**) 200 °C; (**f**) RT and UV irradiation. Reproduced with permission from [44].

Several publications reported the use of ZnO nanowires for gas detection with light activation at room temperature. Procek fabricated ZnO nanostructures containing nanoparticles and nanowires (Figure 22) and used the material for detecting both oxidizing NO_2_ and reducing H_2_, NH_3_. UV illumination enhanced the NO_2_ sensing response at room temperature as high as the effect of thermal activation through heating the sensor to 200 °C. However, light activation was preferred as the sensing response was less sensitive to humidity at lower temperature [44]. The advantage of low temperature operation was also reported by Zhang as mentioned above [51]. Moreover, light activation enhanced selectivity towards NO_2_ while thermal activation enhanced the response towards NH_3_ and H_2_ (Figure 23). By comparing the sensing performance of their devices with those reported in the literature, they concluded that the sensing characteristics were morphology dependent [44]. Similarly, Kiasari reported that both elevated temperature and UV activation would enhance the sensing response of vertically aligned ZnO nanowires towards reducing CO and oxidizing O_2_, but UV illumination was favored because it allowed the sensor to work at room temperature which was less influenced by humidity. They also explained the observed light-activated sensing at room temperature by the popular photon adsorption and desorption model [59].

**Figure 24:**
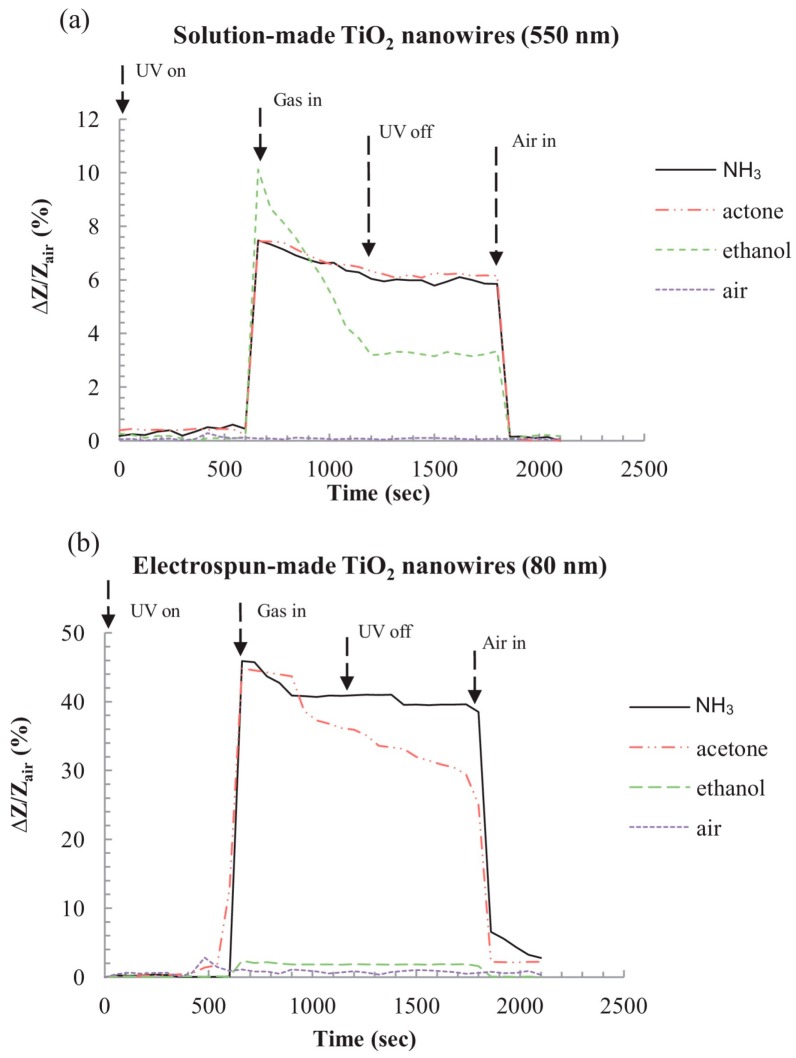
Dynamic response and recovery of sensors based on TiO_2_ nanowires towards vapors with a concentration of 100 ppm: (**a**) sensor based on 2 nanowires synthesized by hydrolysis approach and (**b**) sensor based on 3 nanowires synthesized by electrospinning. Reproduced with permission from [60].

**Figure 25:**
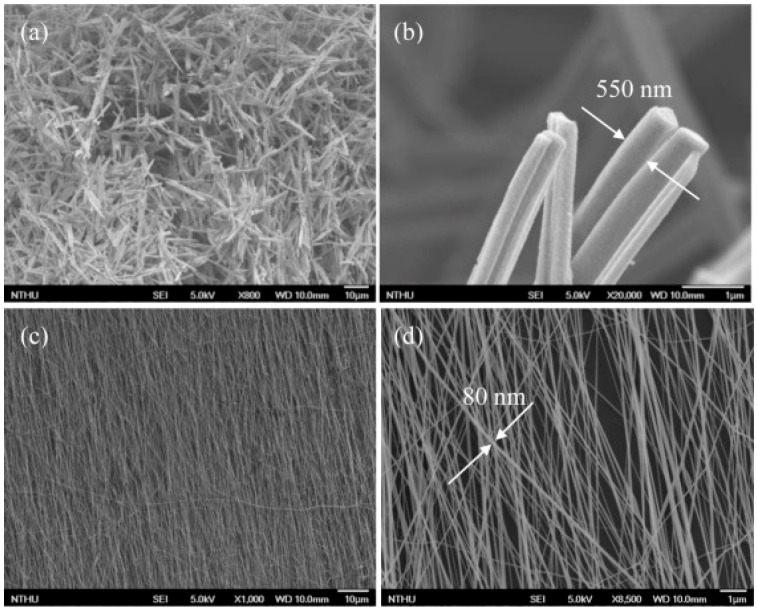
Scanning electron microscopy (SEM) images of TiO_2_ nanowires made from hydrolysis (**a**,**b**) and from electrospinning (**c**,**d**). Reproduced with permission from [60].

The sensing characteristics are dependent on the size of the nanostructures. For 1D nanostructures, the diameter became the most important. Wang fabricated TiO_2_ nanowires with two different methods: ethylene glycol-mediated hydrolysis and high-voltage electrospinning. They compared their sensing response towards NH_3_, acetone and ethanol at room temperature with UV irradiation. It was found that the TiO_2_ synthesized with electrospun exhibited higher response than the one synthesized with solution method (Figure 24). They thought this might be due to the fact that colloidal TiO_2_ nanowires could still contain Ti(OH)_m_ on the surface which would decrease the sensing property because the solution-made TiO_2_ nanowires were fabricated in NaOH solution. However, it was also very possible that the effect was attributed to the smaller diameter of the electrospun-made TiO_2_ nanowires, i.e., 80 nm, and the diameter of the one made from a hydrolysis approach was 550 nm (Figure 25) [60].

**Figure 26:**
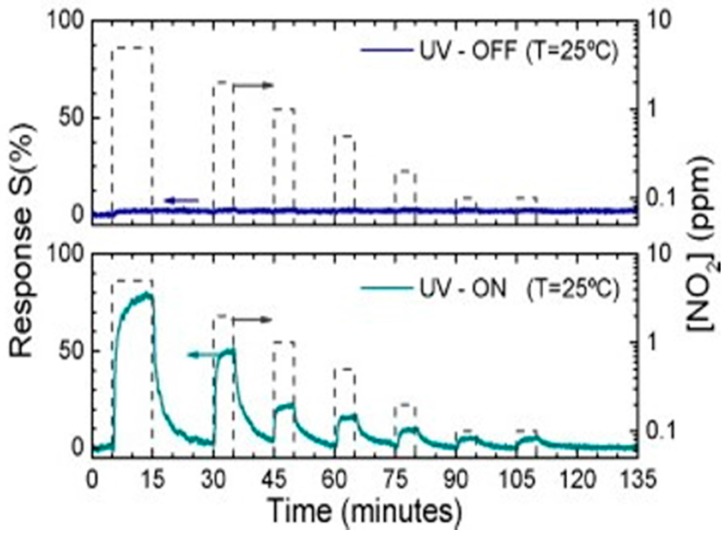
Dynamic NO_2_ sensing curve of an individual SnO_2_ nanowire, operating in dark and UV illumination at room temperature (*T* = 25 °C). Dashed lines indicate the NO_2_ concentration. Reproduced with permission from [39].

**Figure 27:**
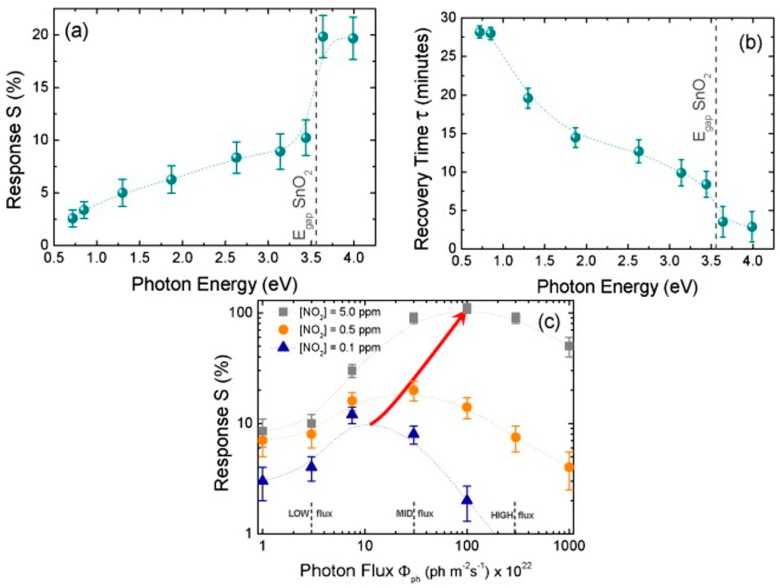
(**a**) Sensor response and (**b**) recovery time of the nanowire device towards 0.5 ppm NO_2_ in air under illumination with different photon energies and constant photon flux; (**c**) Sensor response of three different concentrations of NO_2_ under illumination with different photon flux levels (*E*ph = 3.67 ± 0.05 eV). Reproduced with permission from [39].

Prades used individual SnO_2_ nanowires for NO_2_ sensing at room temperature. The device showed no response in dark and good response under UV light (Figure 26). The observed light-activated NO_2_ sensing at room temperature had strong correlation to photon energy and photon flux. The response and recovery time were at their best levels when the energy of the impinging photons was larger than the bandgap of SnO_2_.The photon flux had also been optimized so that it was large enough to desorb the oxygen species and small enough to prevent massive desorption of NO_2_.They found that the optimal photon flux increased with the increasing of gas concentration (Figure 27) [39,40].

**Figure 28:**
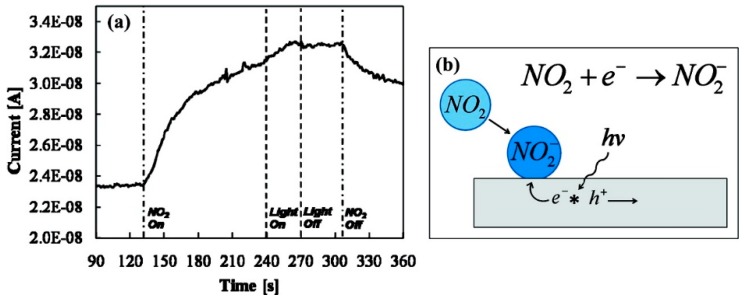
(**a**) The effect of light irradiation on the dynamic response of CuO nanowire (NW) sensor to 100 ppm NO_2_ at room temperature; (**b**) Proposed mechanism. Reproduced with permission from [61].

While most of the efforts have been focused on *n*-type materials, Hansen investigated the light-activated gas sensing of *p*-type CuO for oxidizing NO_2_ and reducing NH_3_. The conductivity of CuO increased upon exposure to NO_2_ at room temperature and increased further with light illumination. However, the conductivity remains steady after removing the illumination, which indicates that the photons increased gas sensing performance and the photon generated electrons participated in the gas molecule adsorption process (Figure 28) [61].

#### 2.2.2. Nanorods

**Figure 29:**
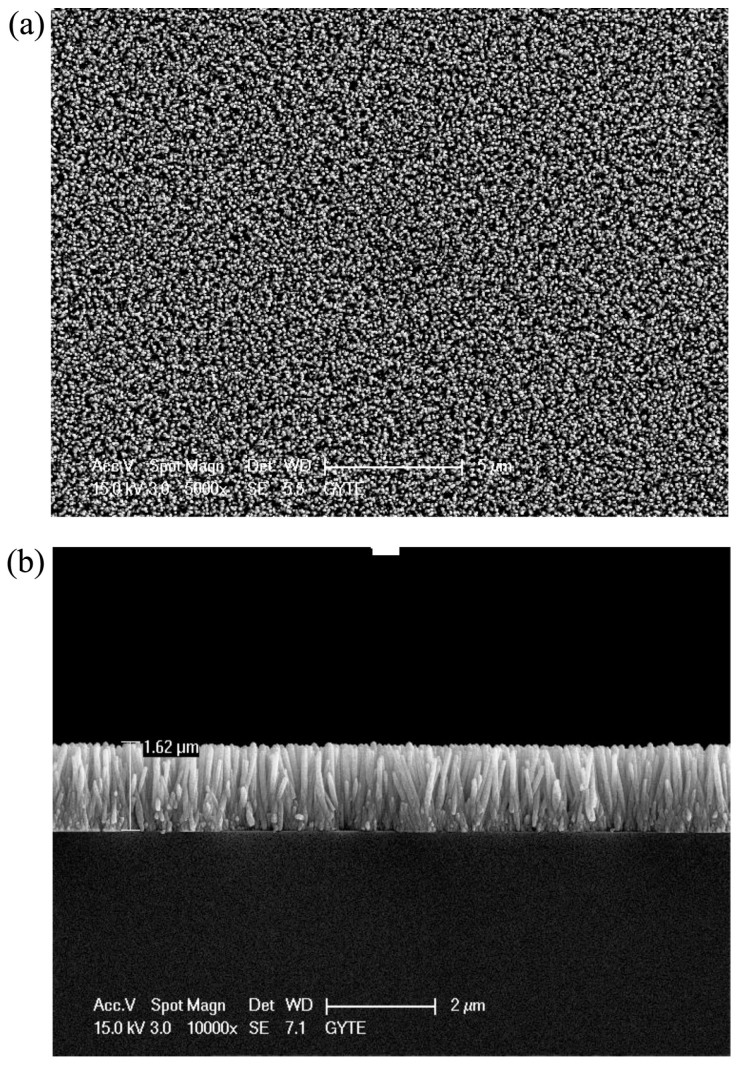
SEM images of ZnO nanorods (**a**) top view and (**b**) cross sectional view. Reproduced with permission from [12].

**Figure 30:**
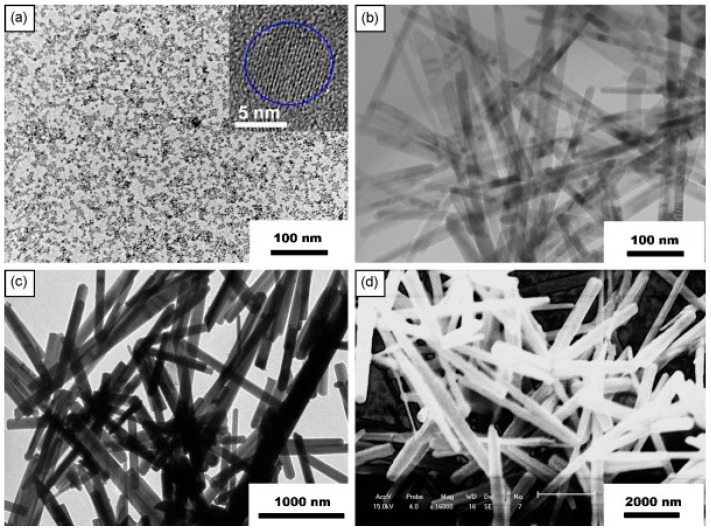
Transmission electron microscope (TEM) of ZnO (**a**) 6 nm nanoparticles (NP-6); (**b**) 40 nm nanorods (NR-40); (**c**) 100 nm nanorods (NR-100); (**d**) SEM of 300 nm ZnO nanorods (NR-300). Reproduced with permission from [13].

**Figure 31:**
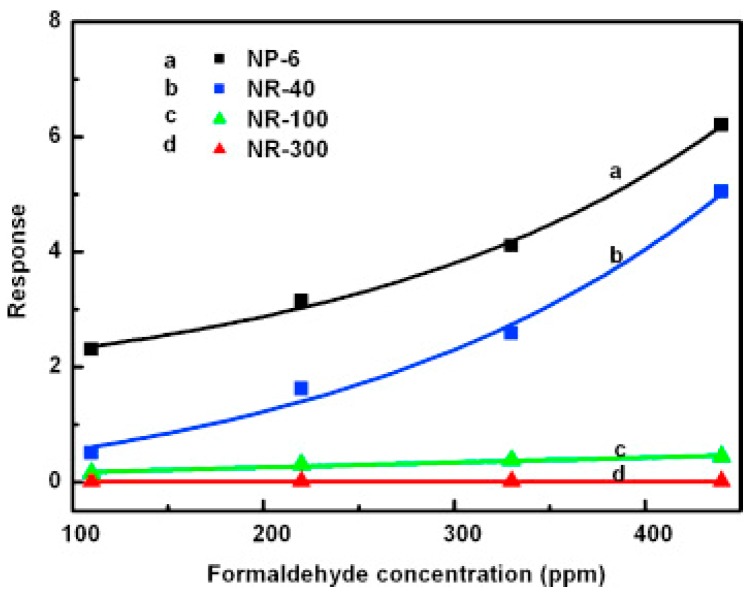
Responses of the NP-6, NR-40, NR-100, and NR-300 samples towards formaldehyde without UV light irradiation. Reproduced with permission from [13].

**Figure 32:**
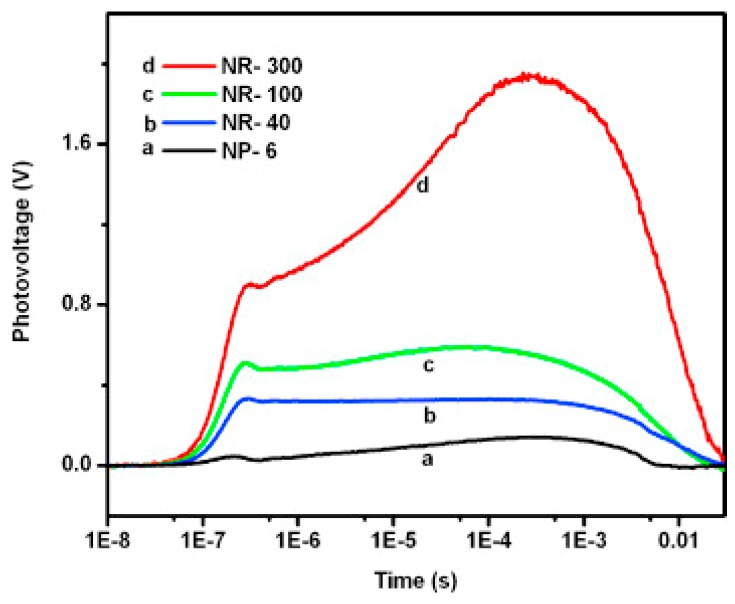
Transient photovoltage (TPV) of (**a**) NP-6; (**b**) NR-40; (**c**) NR-100; and (**d**) NR-300 under 355 nm light irradiation. Reproduced with permission from [13].

Şahin fabricated vertically aligned ZnO nanorods on indium tin oxide coated glass (ITO) and used this material for NO_2_ sensing at 200 °C (Figure 29). White light illumination accelerate the response and recovery process during the NO_2_ sensing. This proposed mechanism behind is the popular photon-induced adsorption-desorption model [12]. Peng studied the size dependence property of ZnO nanorods for sensing formaldehyde in both dark and bright illumination conditions. 6 nm ZnO nanoparticles and nanorods with different diameters (300 nm, 100 nm, 40 nm) were fabricated (Figure 30). Under dark condition, the sensing response increased as the size decrease: 300 nm nanorods < 100 nm nanorods < 40 nm nanorods < 6 nm nanoparticles, as the sensing response was dominated by the surface-to-volume ratio (Figure 31). However, under bright illumination, the 40 nm nanorods exhibited the best sensing response. This was due to photocatalytic reactions, and the sensing response was controlled by both surface-to-volume ratio and photo-generated charge efficiency. Since the photo-generated charge efficiency decreased when the surface-to-volume of the material was increased, which is confirmed by their transient photovoltage (TPV) measurement results (Figure 32), it was important to work out the optimized size to reach a balance for high photo-generated charge efficiency and large surface-to-volume ratio, which was essential for achieving good light-activated gas sensors [13].

#### 2.2.3. Nanofibers

Nanofiber structures have been synthesized and investigated for light-activated gas sensors [14,62]. Gong fabricated ZnO fiber sensors to detect toluene, benzene, acetone and ethanol at room temperature under UV irradiation [14]. Zampetti synthesized titania nanofibers with diameters around 50 nm by electrospinning and evaluated their sensing response towards NH_3_, NO_2_ and humidity at room temperature under UV LED illumination. The nanofibers show better sensing selectivity to NH_3_ and the sensing response is 2.8 towards NH_3_ with very low concentration of 200 ppb [14].

#### 2.2.4. Summary

In this section, we have summarized the one-dimensional nanostructures used in the conductometric gas sensor activated by light. One-dimensional nanostructures including nanowires, nanorods and nanofibers have been reported for gas sensing usage. Whilemost the reported efforts were based on metal oxides nanotubes and nanoribbons, the use of nanotubes and nanoribbons for gas sensor with light activation has received much less attention. It is obvious that this direction should be investigated more.

### 2.3. Metal Oxides with Porous Nanostructures

**Figure 33:**
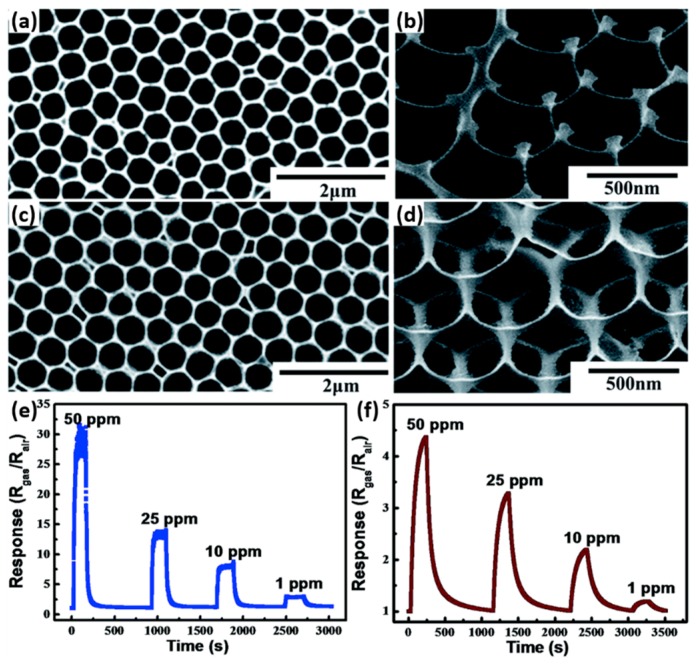
SEM images of (**a**,**b**) bowl-like ZnO micro/nanoporous arrays and (**c**,**d**) close-network ZnO micro/nanoporous arrays. Dynamic response and recovery curve of (**e**) close-network ZnO micro/nanoprous arrays and (**f**) bowl-like ZnO micro/nanoprous arrays towards NO_2_ gas at room temperature under UV light irradiation. Reproduced with permission from [63].

**Figure 34:**
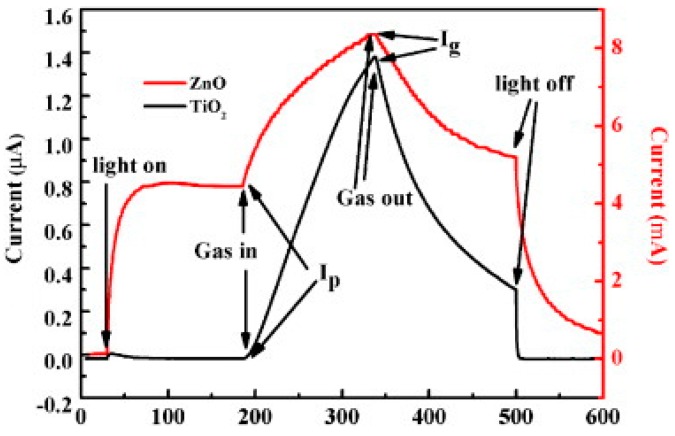
Current transients of TiO_2_ and ZnO after exposure to 75 ppm of formaldehyde gas. Reproduced with permission from [64].

**Figure 35:**
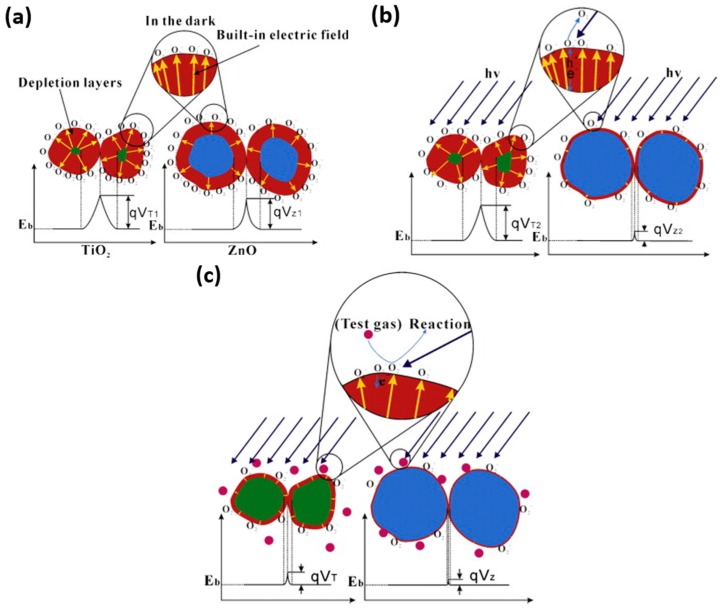
Schematic of surface reactions and corresponding energy barriers (*qV_T_* and *qV_Z_*, in which *q* is the electron charge and *V_T_*, *V_Z_* are potential barriers of TiO_2_ and ZnO, respectively) under different conditions: (**a**) in dry air and in the dark; (**b**) in dry air and with UV light illumination; (**c**) in test gas and with UV light illumination. Reproduced with permission from [64].

Porous structures can enhance gas sensing performance by greatly increasing surface area. A large number of efforts have been conducted to study porous metal oxides. Su used porous array film of ZnO for NO_2_ sensing at room temperature with UV illumination and exhibited very high sensing response. They clarified that the sensing property was highly correlated to the materials morphology by fabricating two nanostructures: close-network micro/nanoporous array film (CNPAF) and bowl-like micro/nanoporous array film (BLPAF) and compared their sensing performance (Figure 33). The CNPAF exhibited much higher sensing response, lower detection limit and faster speed than the BLPAF. The reason was attributed to the presence of closed network which provided higher amount of sensing spots and stronger localization of the flowing gas [63]. The sensing property if also depend on materials. Chen compared the UV light-activated sensing property between porous ZnO and TiO_2_ towards ethanol and formaldehyde at room temperature. Porous TiO_2_ displayed a much higher response to both 100 ppm ethanol (224) and formaldehyde (1700) vapors than ZnO does (0.14 for ethanol and 1.5 for formaldehyde). By comparing their photo response results, they concluded that the material that was less photo-sensitive would be more sensitive to gas (Figure 34). The reason for this conclusion is illustrated in Figure 35. Without light illumination, oxygen molecules were adsorbed on material surface to catch electrons (Figure 35a). After light illumination, electron-hole pairs were generated and the holes would react with the oxygen ions O_2_^−^ to desorb the oxygen molecule (Figure 35b). As shown in Figure 34, compared to TiO_2_, the conductivity of ZnO increased more for a given level of photon flux, which also resulted in more electron-hole pairs being generated, and hence high concentration of O_2_^−^ were oxidized to oxygen molecules and desorbed. As a result, fewer O_2_^−^ remained on the ZnO surface, which led to less interaction with the target gas molecules (Figure 35c) [64].

**Figure 36:**
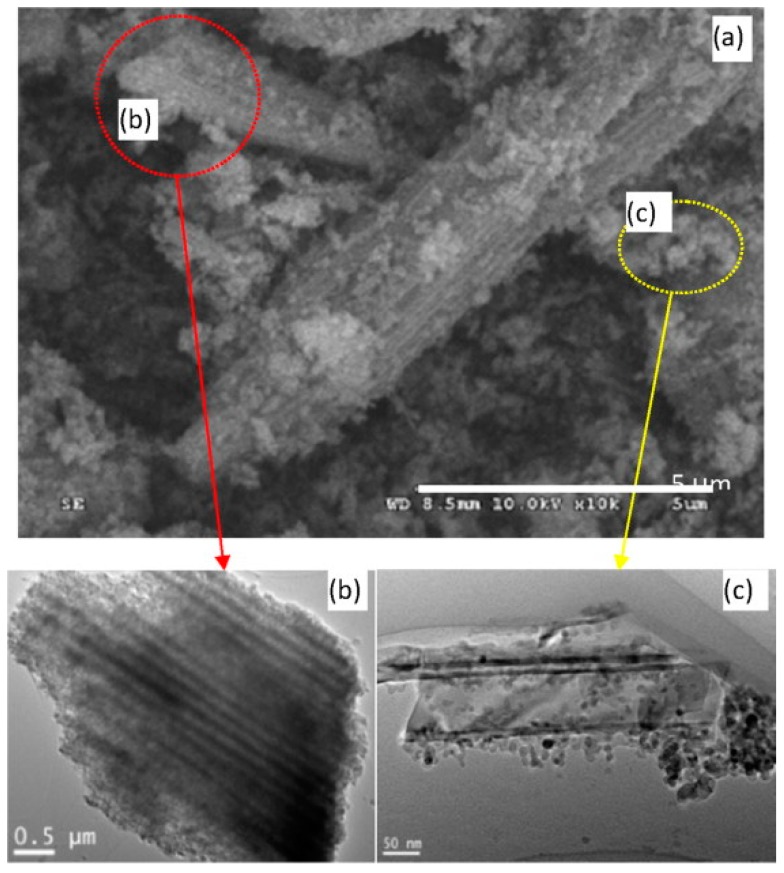
(**a**) SEM images of microporous TiO_2_ and TEM images of (**b**) the larger particles and (**c**) the smaller aggregates shown in (**a**). Reproduced with permission from [65].

**Figure 37:**
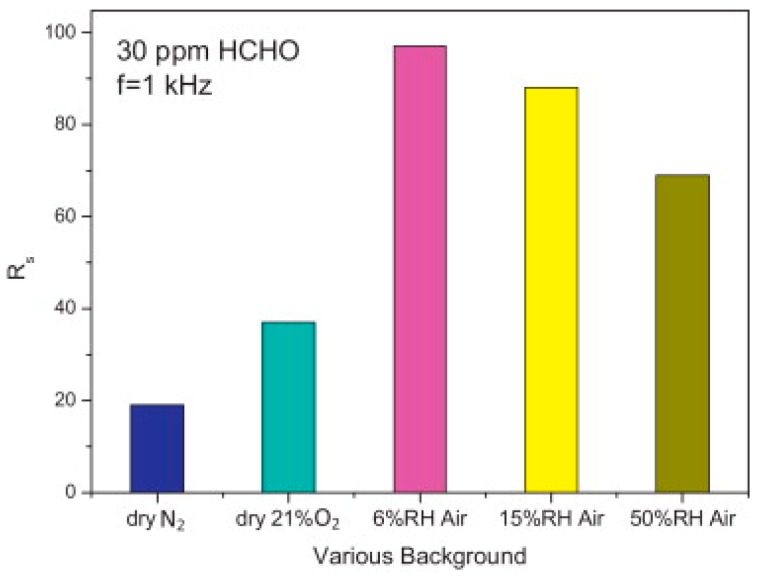
Formaldehyde response of microporous TiO_2_ at room temperature in various background gases. Reproduced with permission from [65].

**Figure 38:**
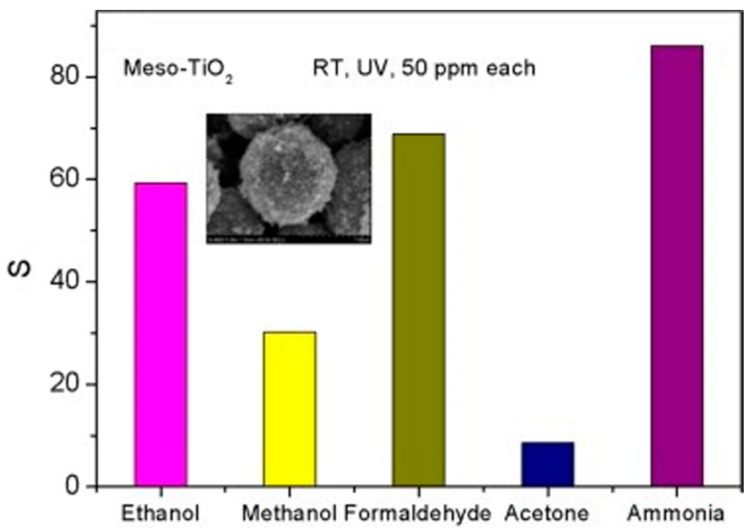
Response of mesoporous TiO_2_ towards various organic volatile compounds (VOCs) in 40% humid air at room temperature with UV illumination. Inset is the SEM of mesoporous TiO_2_ microspheres. Reproduced with permission from [66].

Liu studied the room temperature formaldehyde sensing of microporous TiO_2_ (Figure 36). Instead of the commonly used D.C. resistance measurement, they used an A.C. measurement approach to obtain the impedance which was deemed more stable and with higher response. The sensing response has been defined as the normalized impedance change. They found that UV irradiation together with a certain humidity could improve the formaldehyde sensing response. However, the response decreased with further humidity increasing (Figure 37). This was due to the fact that UV irradiation led to the formation of hydroxyl group from the water adsorbed on the surface of TiO_2_ and hydroxyl would react with interacted formaldehyde molecules which caused the sensing signal. However, if the concentration of water molecules was too high, sensing performance would decrease because they occupied the active adsorption sites and hindered the oxygen species adsorption [65]. Li also fabricated TiO_2_ mesoporous microspheres and demonstrated room temperature sensing towards several organic volatile compounds (VOCs) under UV irradiation in humid air (Figure 38) [66].

**Figure 39:**
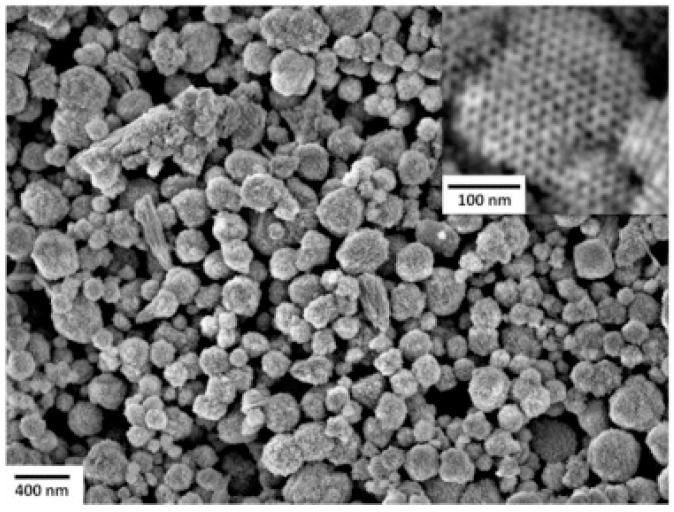
SEM of mesoporous In_2_O_3_. Inset is higher magnification. Reproduced with permission from [68].

**Figure 40:**
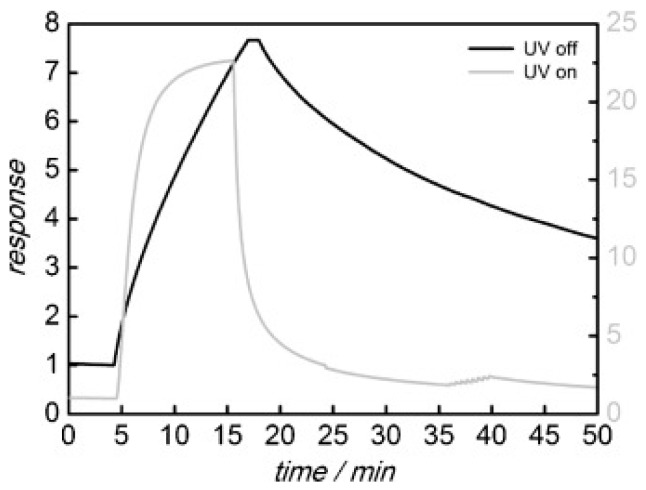
Dynamic response and recovery curve of mesoporous In_2_O_3_ to 5 ppm NO_2_ in dry synthetic air at 50 °C with and without UV illumination. Reproduced with permission from [68].

**Figure 41:**
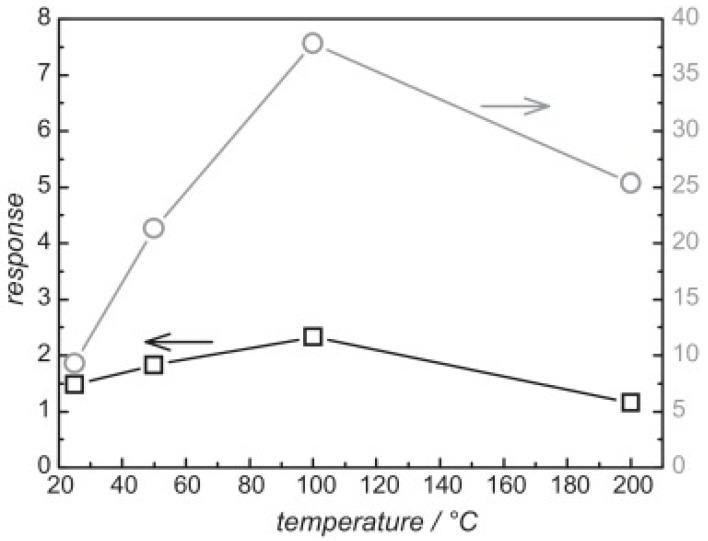
Response towards 5 ppm NO_2_ in dry synthetic air with UV light irradiation at different temperatures of the non-structured (**black**) and the mesoporous In_2_O_3_ (**gray**). Reproduced with permission from [68].

**Figure 42:**
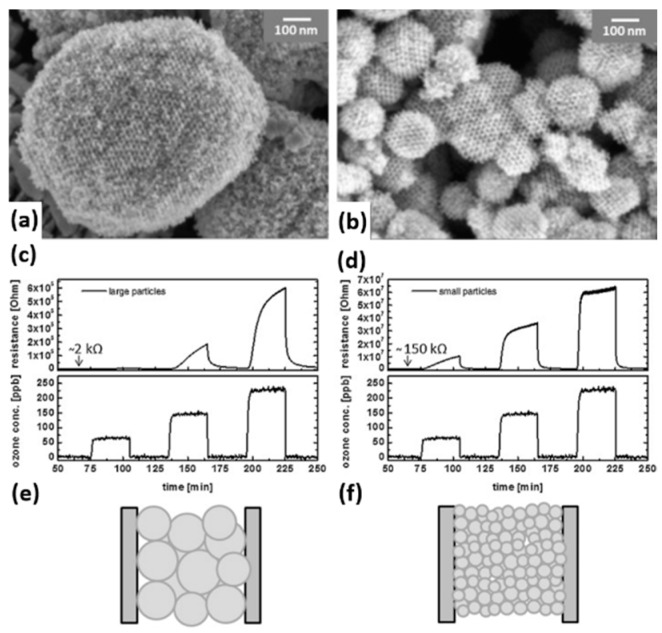
SEM images of ordered mesoporous In_2_O_3_ with large particles (**a**) and small particles (**b**); Dynamic response and recovery to O_3_ at room temperature with blue LED illumination of the large particles (**c**) and small particle layer (**d**); Schemes (**e**,**f**) explaining the difference in the base line resistance for the two sensing layers. Reproduced with permission from [69].

Wagner fabricated mesoporous In_2_O_3_ nanoparticles for NO_2_ detection and compared its sensing property to non-porous In_2_O_3_ (Figure 39) [67,68]. UV irradiation improved the sensing response towards NO_2_ from mesoporous In_2_O_3_ at temperatures lower than 50 °C and accelerated the response and recovery speed by photo-desorption (Figure 40). Under UV irradiation, the mesoporous structure exhibited higher response to NO_2_ than the non-porous In_2_O_3_ did at all temperatures ranging from room temperature to 200 °C (Figure 41). This is attributed to the higher surface-to-volume ratio of mesoporous In_2_O_3_ than the non-porous. Apart from morphology, nanoparticle size also influenced the sensing characteristics. Klaus synthesized ordered mesoporous In_2_O_3_ particles of two different sizes (diameter approx. 170 nm and 870 nm) [69]. The smaller one showed faster sensing speed and lower detection limit to oxidizing ozone with light activation by 460 nm blue LED at room temperature (Figure 42). The faster sensing speed was due to shorter intra-particle distance and thus shorter intra-particle diffusion time in smaller particles. Smaller size resulted in lower detection limit. The sensing response was the resistance change associated with the change of deletion layer thickness in the material, which was caused by the interaction between the sensing material and adsorbed gas molecules. Thus, if the change of the diffusion layer thickness was too small compared to the size of the material upon exposure to a gas of low concentration, the resistance change (i.e., sensing response) would be negligible.

**Figure 43:**
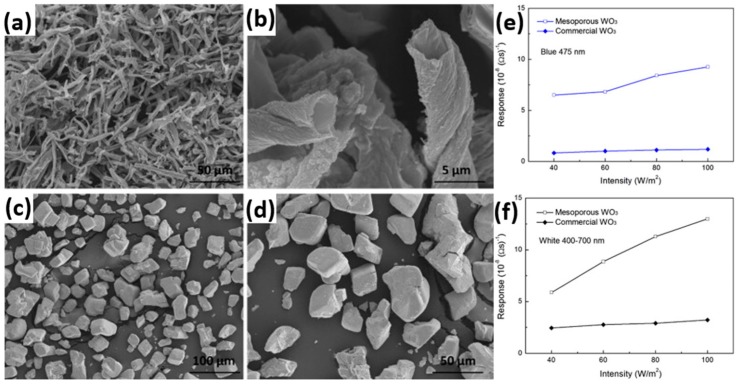
SEM images of the mesoporous (**a**,**b**) and commercial WO_3_ (**c**,**d**); Responses of WO_3_ sensors towards formaldehyde (100 ppm) activated by different light intensity levels; (**e**) Activated by blue light and (**f**) activated by white light. Reproduced with permission from [70].

**Figure 44:**
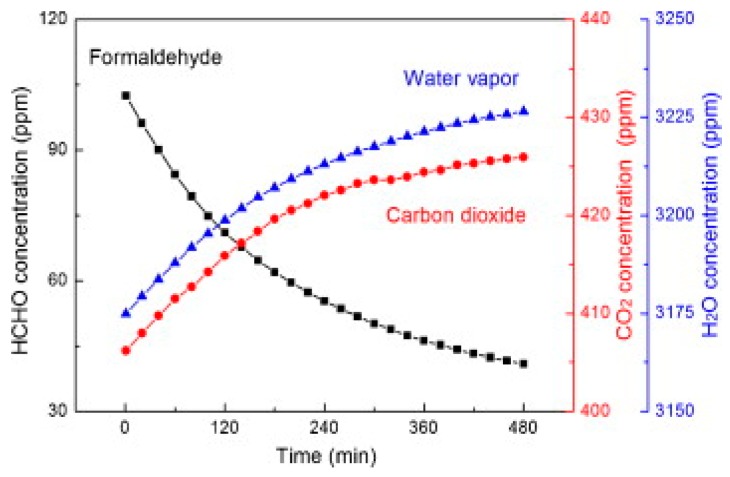
Degradation time vs. concentration of HCHO, CO_2_ and H_2_O. Reproduced with permission from [70].

Deng fabricated mesoporous WO_3_ and compared its sensing property to reducing formaldehyde with commercial WO_3_ [70]. The gas sensing measurement was conducted at room temperature with white light and blue light illumination. Under both white light and blue light irradiation, their mesoporous WO_3_ exhibited higher response than the commercial material did (Figure 43). The enhanced sensing response was attributed to the increased surface area, mesopores and improved light absorption of the mesoporous structure. The increased surface area and mesopores provided more adsorption positions for gas molecules and more reaction sites for the adsorbed oxygen ions and gas molecules. The enhanced light absorption generated more electron-hole pairs which facilitated the production of photon-induced oxygen ions. The response increased linearly with light intensity and gas concentration but decreased significantly in high levels of humidity. They explained the light-activated gas sensing mechanism by the widely accepted photo-adsorption-desorption model. Furthermore, they demonstrated the validity of this photoreaction-induced gas sensing mechanism by measured the concentration of the reactants (i.e., formaldehyde here) and products (i.e., CO_2_ and H_2_O here) (Figure 44).

In this section, the metal oxide materials commonly used in conductometric gas sensors (i.e., ZnO, SnO_2_, TiO_2_, In_2_O_3_ and WO_3_) with porous nanostructure for the light-activated gas sensor are summarized. The advantages of using porous nanostructures are higher surface-to-volume ratio, larger specific surface area and increased light absorption. The effects of varying the structure and size of the material are discussed. Intensity and wavelength of the activation photons and humidity of the atmosphere also play a role in sensing performance. Sensing characteristics of different materials are compared. Generally, smaller size resulted in faster sensing speed and lower detection limit. Porous nanostructures exhibited better sensing performance than non-porous ones. Sensing response may be enhanced by applying a certain level of humidity, but too much humidity can drastically decrease sensing performance.

## 3. Metal Oxide Materials Composites and Metal Oxide with Doping for Gas Sensing

### 3.1. Heterostructures

Heterostructures were reported to offer better gas sensing characteristics in terms of sensing response, speed of response, operation temperature and selectivity as compared to their pure metal oxide counterparts. The enhancement originates from wider light absorption spectrum, increasing carrier concentrations and the benefit of combining the merits of constituent materials [29,32]. Different combinations of materials for the formation of heterostructures have been reported. They include inorganic semiconductors-metal oxides semiconductors, organic semiconductors-metal oxides semiconductors, carbon based materials-metal oxides semiconductors and meta-metal oxides semiconductors. Here, we are focused on combinations between inorganic semiconductors-metal oxide semiconductors and metal-metal oxide semiconductors. Different nanostructures including those synthesized by simple deposition techniques and core-shell are presented.

#### 3.1.1. Heterostructures Consisted of Inorganic Semiconductors and Metal Oxide Semiconductors

##### Simple Coalesced Nanostructure

**Figure 45:**
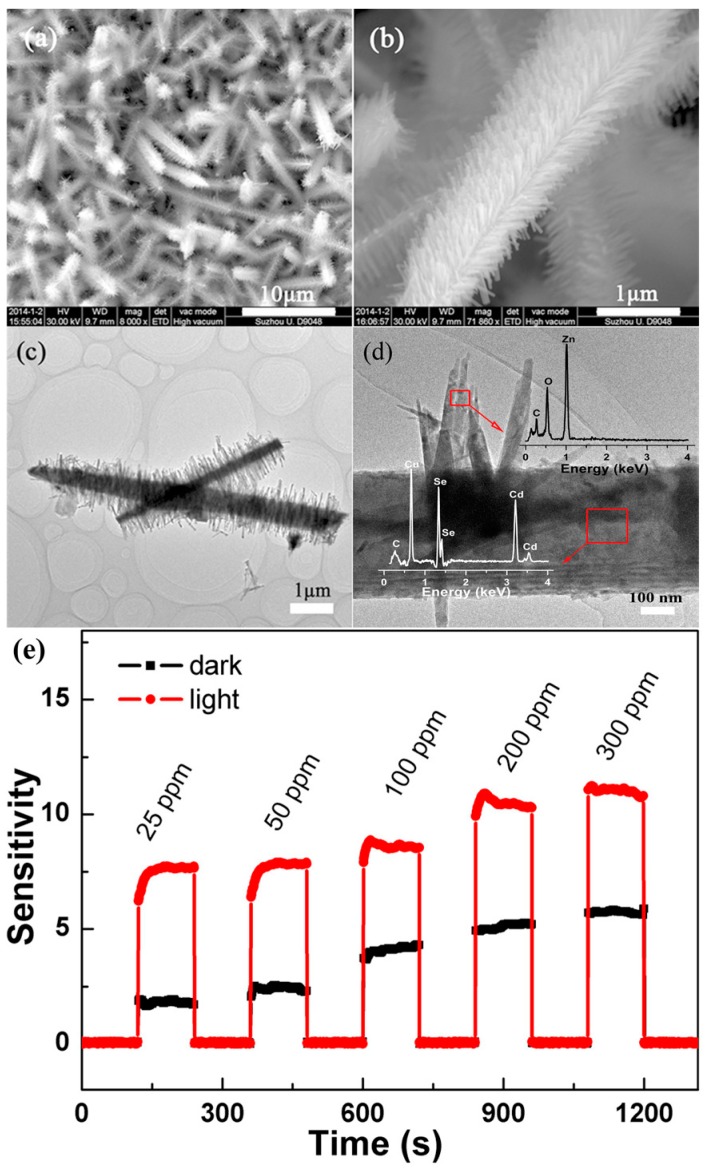
SEM images of (**a**) ZnO nanorods/CdSe nanoribbons heterostructure (HS) and (**b**) an individual ZnO/CdSe HS in high magnification; (**c**) TEM image and (**d**) Energy dispersive X-ray spectroscopy (EDS) spectrum of ZnO/CdSe HS; (**e**) Response of ZnO/CdSe HS-based sensors towards ethanol (25–300 ppm) in the dark and under visible light illumination. Reproduced with permission from [71].

**Figure 46:**
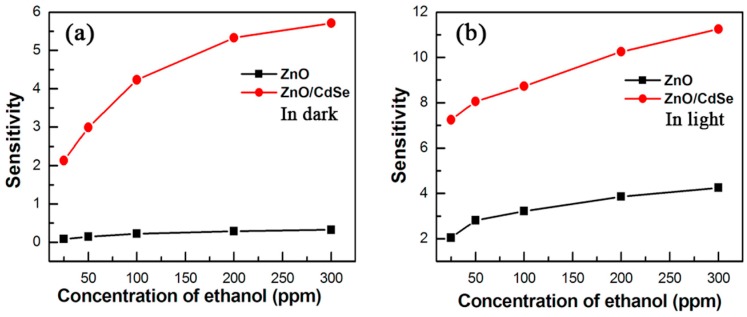
Responses of ZnO nanorod and ZnO/CdSe HS-based sensors to ethanol vapor of different concentrations (**a**) in the dark and (**b**) under visible light illumination. Reproduced with permission from [71].

Wu prepared ZnO nanorods on the surface of CdSe and formed a three-dimensional (3D) ZnO/CdSe heterostructure (Figure 45). Visible light illumination increased the sensing response towards ethanol vapor (Figure 45). This heterostructure lowered the optimal working temperature and exhibited higher response towards ethanol vapors at different concentrations than pure ZnO in both dark and visible light-illuminated conditions (Figure 46). The sensing mechanism was explained with a chemisorption of gas molecules model. The reasons for the improved sensing response for the dark and illuminated cases are different. In dark, for the heterostructure, a depletion layers are formed between the two materials because of their different Fermi levels. In this depletion region, electrons are easily released and oxygen molecules are more ready to be adsorbed, resulting in better sensing outcome. With visible light illumination, electron-hole pairs are generated in CdSe because of its narrow bandgap (1.7 eV). These photoelectrons were injected into the conduction band of ZnO due to the Fermi level difference between CdSe and ZnO, thus suppressing the recombination of photon generated electron-hole pairs in CdSe and hence leading to higher concentration of energetic electrons [71].

**Figure 47:**
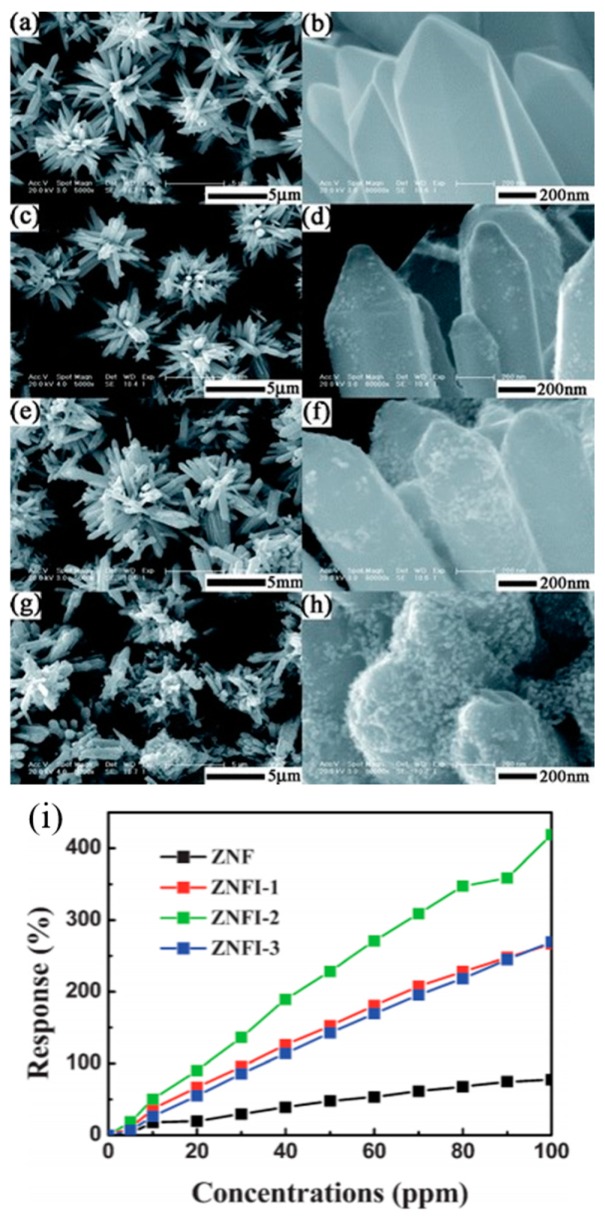
SEM images of ZnO and In_2_O_3_-sensitized ZnO nanoflowers: (**a**,**b**) pure ZnO nanoflowers; (**c**,**d**) ZNFI-1; (**e**,**f**) ZNFI-2; (**g**,**h**) ZNFI-3; (**i**) HCHO responses of the ZnO and In_2_O_3_-sensitized ZnO nanoflowers under 460 nm photon irradiation. Reproduced with permission from [72].

Han fabricated In_2_O_3_ sensitized ZnO flowers and studied their formaldehyde gas sensing property under visible light irradiation at room temperature. This heterostructure exhibited photoresponse to visible light due to the In_2_O_3_ sensitization. As shown in Figure 47, adding In_2_O_3_ benefited the HCHO sensing at room temperature under 460 nm light illumination because of the increased carrier concentration. Heterostructures improved the utilization of photon generated electron-hole pairs by suppressing their recombination. Apart from carrier concentration, the sensing performance was also dependent on surface area. Thus, too much In_2_O_3_ might be seriously agglomerated on the ZnO surface and the density of active sites might be decreased [72].

**Figure 48:**
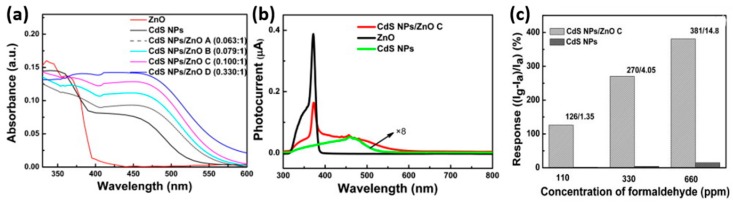
(**a**) UV-vis diffuse reflectance spectra; (**b**) photocurrent of ZnO, CdS NPs and the CdS NPs/ZnO A, B, C and D (A, B, C and D: mass ratios of CdS/ZnO were 0.063:1, 0.079:1, 0.100:1 and 0.330:1, respectively); (**c**) Formaldehyde sensing response under visible light illumination of CdS and CdS NPs/ZnO C. Reproduced with permission from [10].

Zhai sensitized commercial ZnO nanocrystals with CdS nanoparticles for formaldehyde sensing at room temperature under visible light illumination. Heterostructures of different mass ratios (CdS: ZnO) were prepared and their sensing performance were compared. As shown in Figure 48a, ZnO decorated with CdS expanded the absorption spectrum to the visible range after 400 nm, while pristine ZnO only absorbed light at wavelengths shorter than 400 nm. This CdS sensitization effect was confirmed by the photocurrent results (Figure 48b). The CdS decoration significantly enhanced room temperature HCHO sensing response of ZnO activated by light as shown in Figure 48c. The improvement of the heterostructure was dependent on the mass of ratio of CdS and ZnO. The CdS/ZnO with 0.1:1 exhibited the highest sensing response to both HCHO with both high and low concentration levels. Further increasing the concentration of CdS decreased the sensing response. This was because increasing the CdS content on ZnO surface could both increase the photocarrier concentration and decrease the effective surface area of ZnO. This phenomenon and explanation is highly accepted [10].

**Figure 49:**
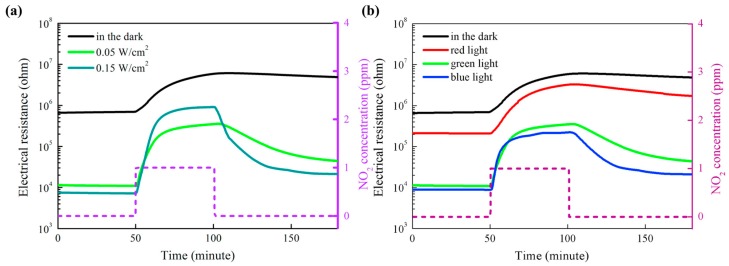
Dynamic sensing plots of Cds-ZnO to 1 ppm NO_2_ at room temperature vs. (**a**) green light intensity (0, 0.05 and 0.15 W/cm^2^); (**b**) different light wavelength. Reproduced with permission from [73].

**Figure 50:**
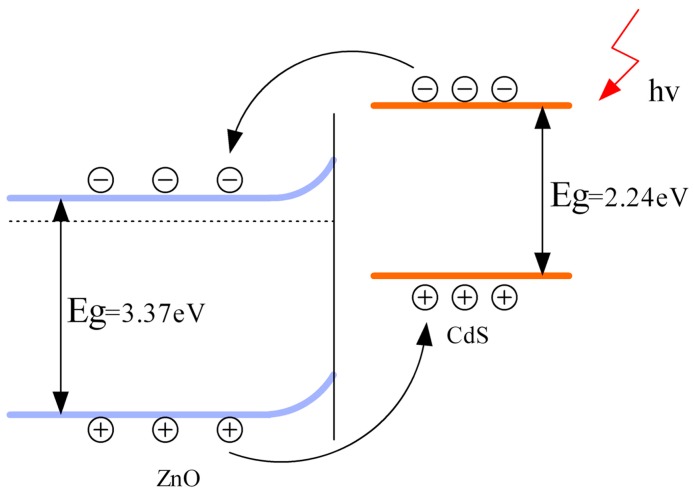
Schematic of carrier transportation in CdS-ZnO under visible light. Reproduced with permission from [73].

There are also reports on the use of heterostructures for sensing oxidizing gases [73,74]. Geng fabricated porous CdS-ZnO for sensing NO_2_ at room temperature with light illumination at different wavelengths. They showed that the CdS worked as a sensitizer which enabled visible light activation. They also studied the relationship between sensing response and light intensity, light wavelength and material structure (Figure 49). The sensing response of CdS-ZnO to NO_2_ was enhanced with green light illumination and further increase by increasing the light intensity (Figure 49a). The sensing speed was also accelerated by increasing light intensity. The NO_2_ sensing properties were also compared under light with different wavelengths while maintaining power constant and green light produced the highest sensing response, which was consistent with the light absorption spectrum of the sample (Figure 49b). The CdS-ZnO exhibited higher response to NO_2_ than pure ZnO under green light illumination and further increase in sensing response was achieved by introducing more pores in the CdS-ZnO composite. The mechanism of gas sensing in dark and with light illumination was attributed to the widely accepted surface depletion model. Three factors were influencing the gas sensing properties: carrier concentration, depletion region width and grain boundary barrier height. The improved sensing performance from CdS-ZnO heterostructures were associated with both the CdS sensitization and the electron injection from CdS to ZnO, which suppressed the recombination of photogenerated electron-hole pairs and lengthened electron lifetime (Figure 50) [73].

Hoffmann also fabricated CdS-ZnO on Si substrate for sensing of various gases including both oxidative and reducing gases at room temperature with solar light adsorption. Though this gas sensor was based on measurement of voltage change, which was different from conductometric case, its sensing mechanism was the same surface depletion model. The CdS-ZnO only exhibited response to both O_2_ and ethanol with light illumination which was due to the presence of photon-electrons. The CdS-ZnO exhibited higher response than pure ZnO to methane with light illumination and the improvement by incorporating a heterostructure was due to the increase of electron concentration, as mentioned above [75].

**Figure 51:**
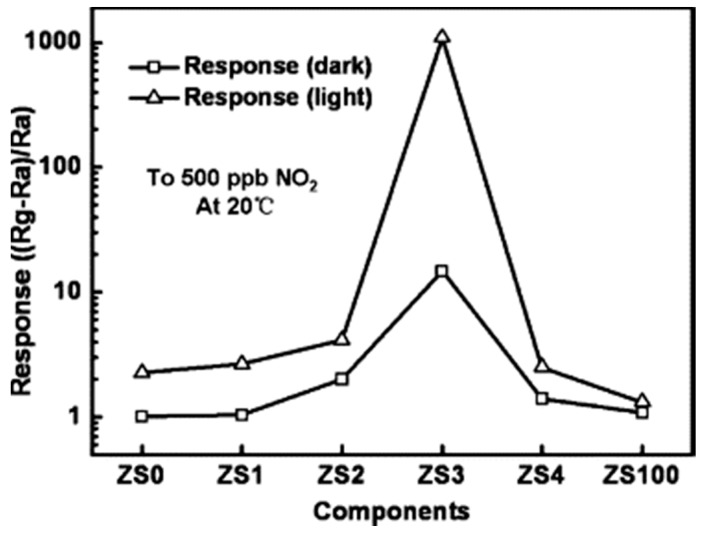
NO_2_ sensing response at room temperature in dark and under UV light irradiation of ZnO/SnO_2_ heterostructure with different ZnO: SnO_2_ molar ratios. Reproduced with permission from [74].

**Figure 52:**
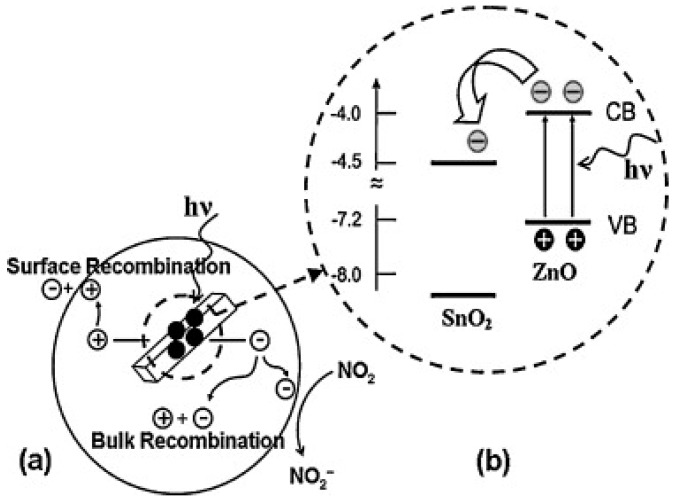
Schematic diagram: (**a**) UV stimulated carriers transport; (**b**) energy band structure and electron-hole pair separation and transportation in the ZnO/SnO_2_ heterostructure in the area marked with a dashed circle. Reproduced with permission from [74].

**Figure 53:**
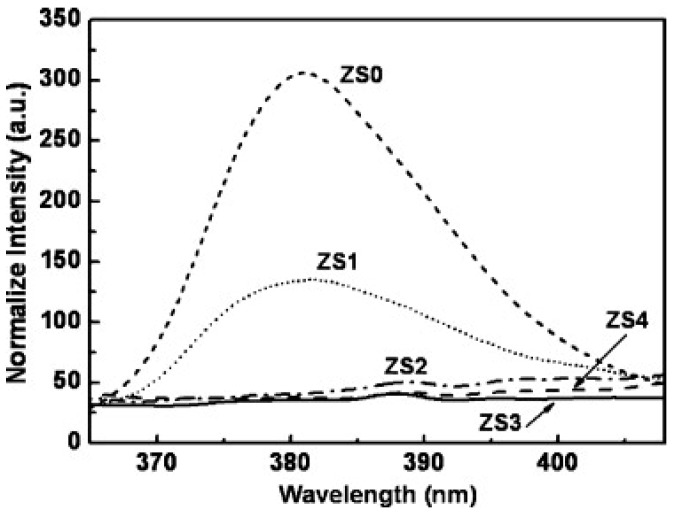
Photoluminescence (PL) emission spectra of ZnO/SnO_2_ heterostructure with different ZnO: SnO_2_ molar ratio. Reproduced with permission from [74].

Different from Geng’s work, which used ZnO as the electron acceptor under visible light illumination, Lu used ZnO as an electron donor under UV illumination by fabricating ZnO/SnO_2_ heterostructures. The ZnO/SnO_2_ heterostructure was used for detecting several oxidizing and reducing gases at room temperature with and without UV illumination. UV illumination improved sensing response for all of the gas types because photo-generated electrons facilitated gas adsorption and desorption. Selectivity and sensing speed were also improved by UV illumination. Compared to both pure ZnO nanorods (ZS0) and pure SnO_2_ nanoparticles (ZS100), the ZnO: SnO_2_ heterostructures all exhibited higher sensing response to NO_2_ in both dark conditions and UV irradiation conditions (Figure 51). The photo-generated electrons-hole pairs showed short lifetime because of their recombination in pure semiconductors, whereas the heterostructure increased electron concentration and lifetime by suppressing the recombination through injected UV generated electrons from ZnO to SnO_2_ due to their different Fermi levels (Figure 52). This model was confirmed by photoluminescence (PL) which indicated the increased electron concentration in ZnO/SnO_2_ heterostructures (Figure 53). They also investigated how ZnO: SnO_2_ ratio would affect the sensing performance and found that a ratio of 1:1 was optimal for achieving the highest response towards NO_2_ under UV illumination. Generally, increasing the ratio of SnO_2_ would benefit sensing response because electrons were accumulated in SnO_2_. However, too much SnO_2_ would lead to too much coverage of the ZnO surface, which in turn blocked a large portion of light adsorption sites and finally decreased the concentration of photo-generated electrons. Thus, too much SnO_2_ would degrade sensing performance. Nonetheless, the 1:1 ZnO/SnO_2_ molar ratio also matched the Brunauer-Emmett-Teller (BET) Surface Area Analysis which indicates that the 1:1 ZnO/SnO_2_ heterostructures contained the largest surface area. Increased humidity decreased the response both in dark and under UV illumination [74].

The visible sensitization effect of heterostructures was observed by several other teams. For example, Yang sensitized ZnO nanoparticles with Ru(dcbpy)_2_(NCS)_2_ for CO and O_2_ sensing under visible light illumination at room temperature. The light source was of 545 nm wavelength, which matched with the absorption peak of Ru(dcbpy)_2_(NCS)_2_-sensitized ZnO [76].

**Figure 54:**
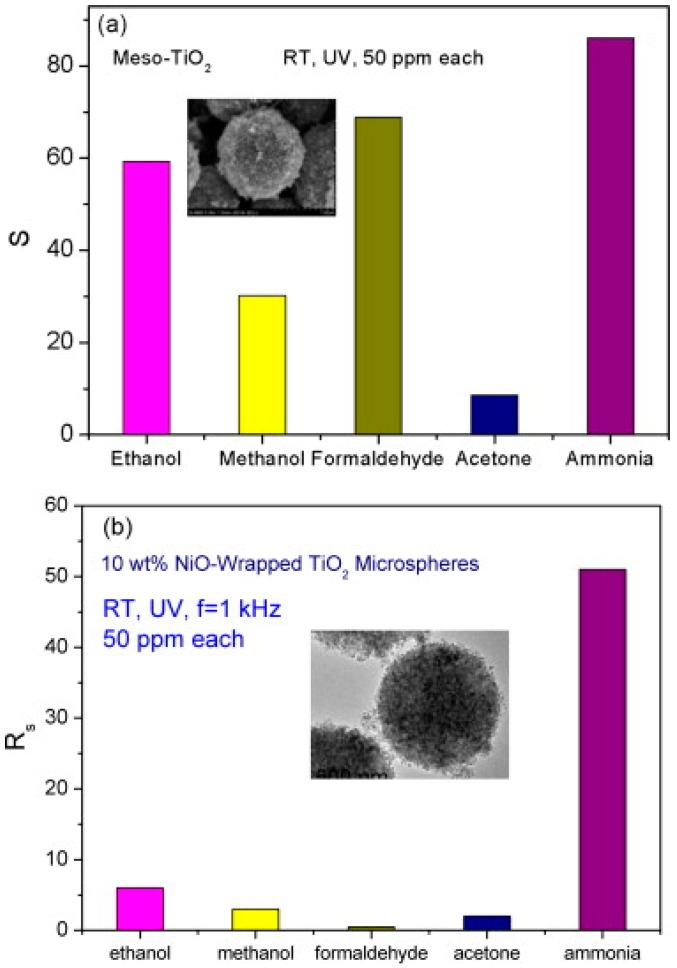
Sensing response of VOCs (**a**) pure meso-TiO_2_ and (**b**) 10 wt % NiO-wrapped TiO_2_ microspheres based sensors in humid air (RH ~ 40%) under UV illumination at room temperature. Reproduced with permission from [66].

In addition to the enhancement in sensing response and sensing speed due to the expanded light absorption and increased electron concentration, selectivity could be improved by combining the sensing merits of different materials. Li wrapped NiO on mesoporous TiO_2_ and compared its sensing characteristics with those of pure mesoporous TiO_2_. The materials were used for sensing various gases at room temperature under UV illumination. Pure TiO_2_ shows response to all the gas samples while NiO-TiO_2_ only exhibited high response to NH_3_ (Figure 54). The enhanced selectivity was attributed to the cancelling sensing characteristics between *p*-type NiO and *n*-type TiO_2_ [66].

**Figure 55:**
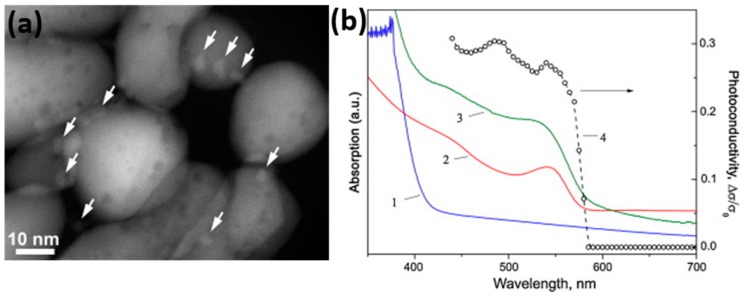
(**a**) High angular annular dark field scanning transmission electron microscopy (STEM) image of CdSe QD1/ZnO sample. CdSe nanoparticles are marked with arrows; (**b**) Absorption spectra of ZnO powder (1); CdSe QDs suspended in solution (2); QD3/ZnO powder (3); and dependence of photoconductivity of QD3/ZnO sample on the excitation wavelength (4). Reproduced with permission from [78].

Apart from nanoparticles decoration, some publications reported the use of quantum dots (QDs) to enhance the sensing performance of metal oxides. Chizhov loaded CdSe quantum dots on several kinds of metal oxides including ZnO, SnO_2_ and In_2_O_3_ [77,78]. Figure 55a shows the scanning transmission electron microscopy (STEM) images of the CdSe QDs sensitized ZnO. The CdSe QDs sensitized these metal oxides to absorb visible light, (Figure 55b) thus enabled visible light-activated NO_2_ sensing at room temperature [77]. The improved sensing response under visible light illumination was due to the increased carrier concentration in metal oxide, which was injected from the CdSe QDs. They also found that the most effective sensitization was achieved for In_2_O_3_ because of the maximum band offset between In_2_O_3_ and CdSe QDs [78].

##### Core-Shell Nanostructure

**Figure 56:**
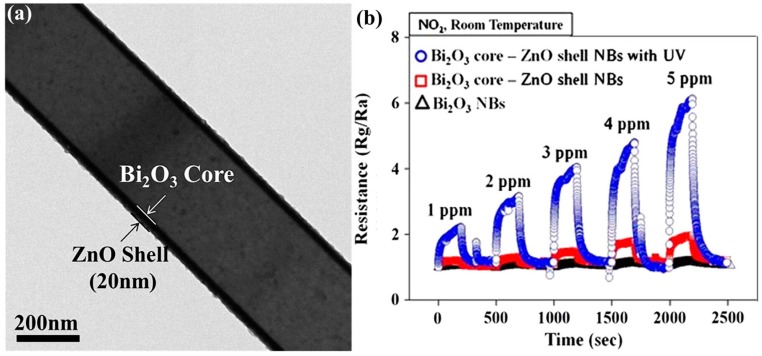
(**a**) Transmission electron microscopy (TEM) image of a Bi_2_O_3_-core/ZnO-shell nanobelt; (**b**) Dynamic NO_2_ sensing plot of pure Bi_2_O_3_ nanobelts in dark, Bi_2_O_3_-core/ZnO-shell nanobelt in dark and with UV illumination. Reproduced with permission from [80].

In addition to the coalesced structure, a special core-shell heterostructure has also been studied [41,79,80]. Park synthesized Bi_2_O_3_-core/ZnO-shell nanobelt for NO_2_ sensing at room temperature (Figure 56). The ZnO coating enhanced response to NO_2_ of pristine Bi_2_O_3_ without light illumination was due to the suppressed recombination and facilitated electrons transportation. The sensing response was further improved with UV illumination due to the photo-generated electrons (Figure 56) [80]. They also used ZnO as the core and synthesized ZnO-ZnS core/shell nanowires to detect reducing ethanol vapors at room temperature with UV illumination. The ethanol sensing performance was compared between the ZnO core/ZnS shell and pure ZnO nanowires, and their results showed improved sensing performance. The improvement was caused by the formation of an energy barrier at the ZnO-ZnS interface [79].

**Figure 57:**
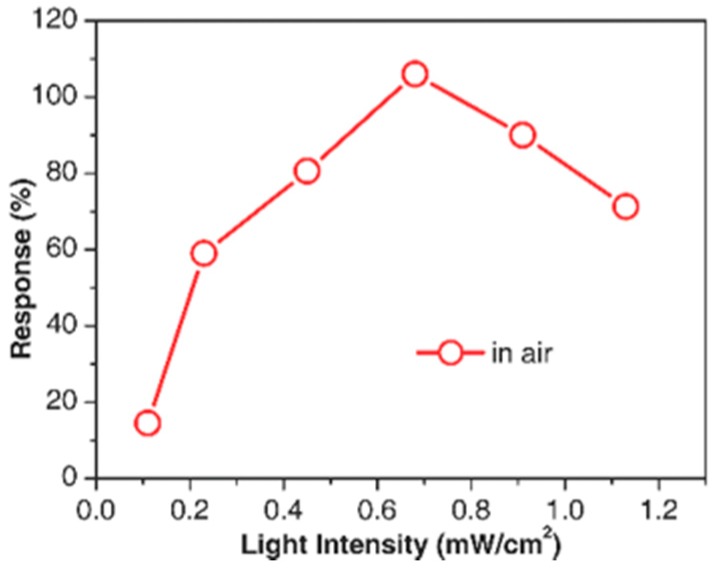
NO_2_ sensing response of CdS/ZnO core/shell nanowires under illumination of different light intensity. Reproduced with permission from [41].

Yang fabricated CdS/ZnO core/shell nanowires and assembled them to form a photodetector and a NO_2_ optoelectronic sensor. The sensing of NO_2_ was activated by visible light due to the sensitization effect of CdS as mentioned before. They compared the photocurrent under 468 nm light illumination between CdS core/ZnO shell and pristine CdS, the heterostructure showed a remarkable enhancement in photoresponse. This improvement indicated that heterostructure increased carrier concentration by inhibiting recombination. They also investigated how light intensity influenced the NO_2_ sensing response. The sensing response does not keep increasing as the light intensity increases. Instead, there was an optimal light intensity at which the sensing exhibited maximized response (Figure 57). The optimal light intensity balanced the increased photo-carriers, which improved the gas sensing property, and the photo-induced gas desorption [41].

#### 3.1.2. Heterostructures Containing Metal and Metal Oxide Semiconductors

Apart from inorganic-semiconductor/metal oxides, metal/metal oxide heterostructures have been investigated in the field of light-activated conductometric gas sensor. Common metals including Au, Ag, Pt and Pd, as well as Al, were used to decorate metal oxides. Karaduman used Al/TiO_2_/Al_2_O_3_/p-Si for sensing oxidizing NO_2_ and the response at room temperature was only achieved with UV irradiation [81]. Han studied H_2_ sensing performance of Pt/Pd/TiO_2_/Al_2_O_3_ at different temperatures. They found that UV irradiation lowered the optimal working temperature from 253 °C to 82 °C and accelerated sensing speed [82]. Deng fabricated a gas sensor based on Pt-modified ZnO to improve sensing selectivity. They compared the gas sensing selectivity among different operation modes. The sensor working at a constant temperature exhibited a broad-spectrum response to multiple analytes with poor selectivity. Different methods including simple temperature modulation and simple light modulation to enhance the gas sensing selectivity. Temperature modulation could improve the selectivity by modify the adsorption and desorption kinetics and the light modulation could generate photocarriers for the sensing process. In their work, they claimed that the combination of temperature and light modulation offered the best gas selectivity of Pt modified ZnO sensor [83]. Peng synthesized copper decorated ZnO (1 mol %) nanocrystals by sol-gel method and used it for ethanol and acetone sensing at room temperature. They compared the response of Cu/ZnO heterostructures in conditions with and without 355 nm UV illumination. The illumination improved sensing response to both ethanol vapors and acetone vapors due to the photocatalysis and photoadsorption effect [11]. Elisabetta Comini synthesized SnO_2_ with different materials decoration including Au, In and Si for CO sensing at different temperatures, UV illumination improved the sensing response more at lower working temperature than at high temperature [84]. Chen synthesized Pt/TiO_2_-WO_3_ heterostructures for ozone monitoring under visible light irradiation and found that Pt decoration promoted the ozone sensing response and recovery under 460 nm light irradiation. This improvement was attributed to the increased separation of electron-hole pair and decreased recombination of electron-hole pairs caused by charge transferring among materials [85]. Fahimeh Hooriabad Saboor demonstrated that a small amount of Pd loading could enhance NO_2_ sensing response and decrease the recovery time of SnO_2_ under UV light irradiation at room temperature. Different annealing temperatures and nanostructures influenced the materials sensing performance. The UV light intensity also affected the NO_2_ response and different materials exhibited different optimal UV light intensity [21]. Trawka decorated WO_3_ nanowires with Au nanoparticles to detect oxidizing NO_2_ and reducing ethanol, and found that UV irradiation could enhance sensing response and lower working temperature [54]. They used the same heterostructure to study its sensing property to ethanol, methane and formaldehyde and found the UV irradiation could improve the sensing response and the sensing selectivity could be controlled by tuning the light wavelength [55].

**Figure 58:**
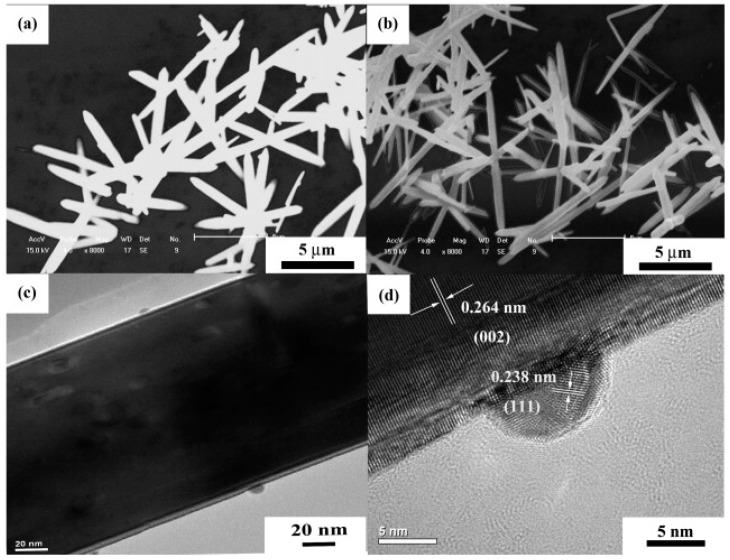
SEM images of (**a**) pure ZnO nanorods and (**b**) Ag-ZnO nanorods-3; (**c**) TEM image and (**d**) High-resolution transmission electron microscopy (HRTEM) image of Ag-ZnO nanorods-3. Reproduced with permission from [86].

**Figure 59:**
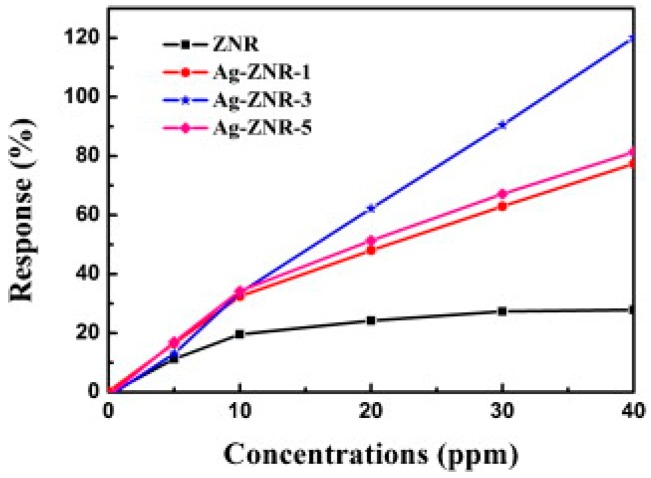
HCHO sensing response of ZnO and Ag-ZnO nanorods under 370 nm irradiation. Reproduced with permission from [86].

There are a few papers in the literatures on studying the gas sensing improvement by incorporating metal decoration. Cui synthesized Ag-ZnO nanorods with enhancement under UV light-activated formaldehyde sensing at room temperature (Figure 58) [86]. Different amounts of Ag decorating were studied. The formaldehyde sensing response increased initially with the increasing of Ag amount but decreased then when too much Ag was decorated (Figure 59). The improved effect of Ag NPs was attributed to two mechanisms: (1) Ag nanoparticles facilitated the oxygen adsorption and dissociation; (2) The interaction between Ag nanoparticles and ZnO facilitated the photoelectron separation and transportation, thus suppressed the recombination of photogenerated electron-hole pairs. This improvement mechanism was confirmed by their surface photovoltage and phase spectra study which indicated that photoelectrons transported to the ZnO surface and captured by Ag nanoparticles. Too much Ag nanoparticle decoration would result in over-covering the ZnO surface, which then led to reduction in the density of active reaction sites, thus decreasing the sensing performance.

Similar results were observed by Dhahri who synthesized Al loaded ZnO for CO sensing. The sensing performance was assessed by operating the device in dark and under UV light illumination at different temperatures. While UV illumination enhanced the sensing response and accelerate the recovery process at all temperatures, Al loading exhibited higher CO sensing response than pure ZnO did at different working temperatures with UV illumination. The sensing response was dependent on the amount of Al, though they did not analyze this in their paper [87].

**Figure 60:**
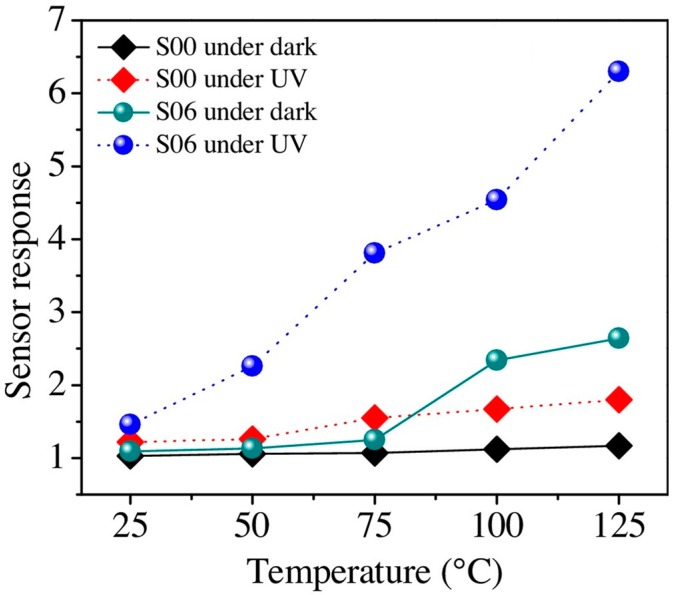
1000 ppm ethanol sensing response of bare ZnO (S00) and Au NPs decorated ZnO at the sputtering time of 6 s (S06) sensors under dark condition and UV illumination with intensity at 4.1 mW/cm^2^. Reproduced with permission from [88].

**Figure 61:**
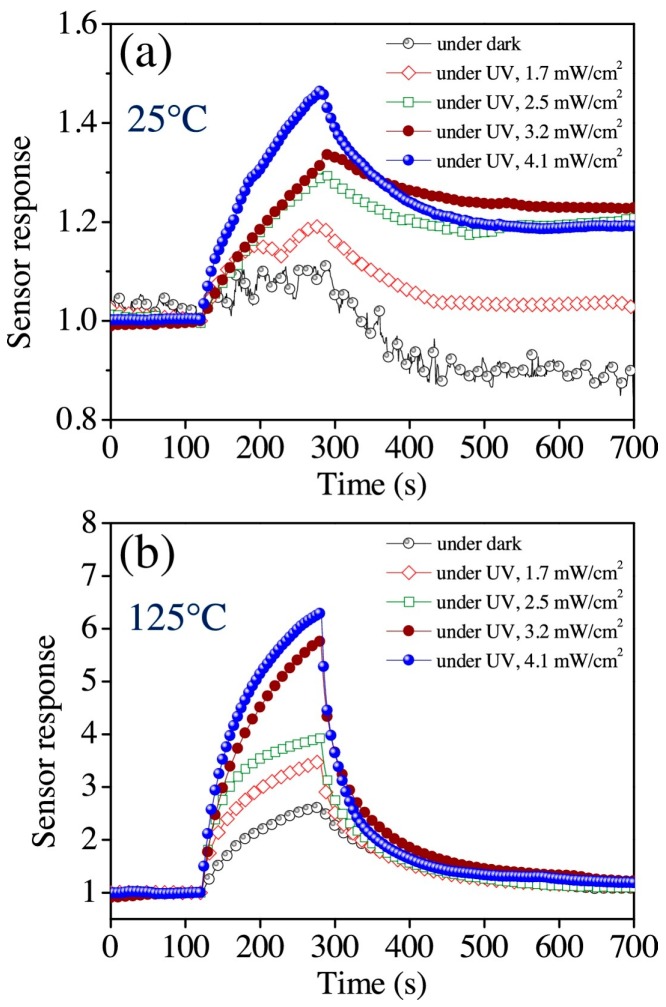
Dynamic 1000 ppm ethanol vapor sensing plots of S06 sensor with different UV illumination intensities operated at operating temperatures of (**a**) 25 °C and (**b**) 125 °C. Reproduced with permission from [88].

**Figure 62:**
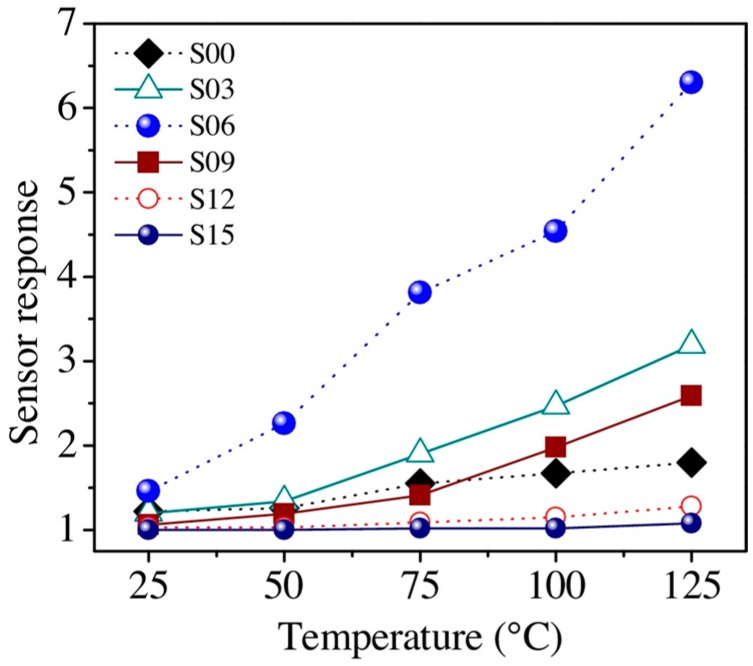
1000 ppm ethanol sensing response of pure ZnO (S00) and ZnO decorated with Au NPs of different amount (S03–S15) under UV illumination intensity of 4.1 mW/cm^2^. Reproduced with permission from [88].

**Figure 63:**
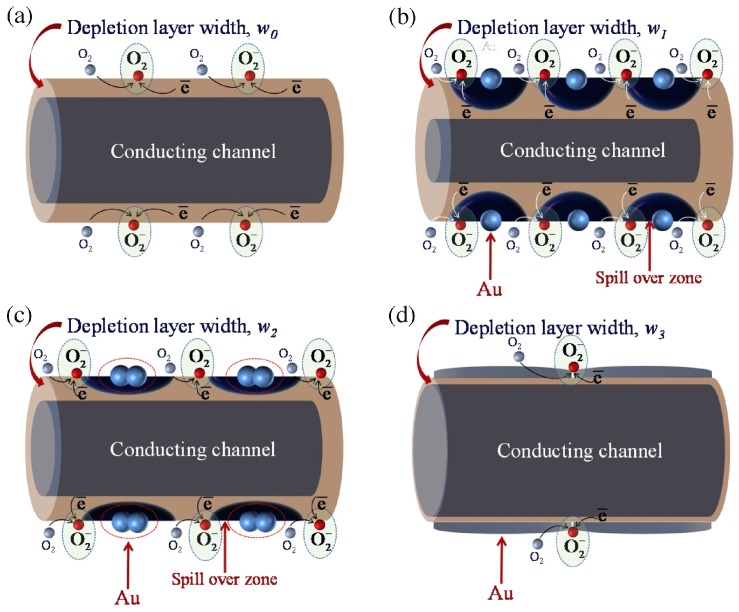
Schematic diagrams of depletion layer width resulting in sensor response of ZnO nanostructures sensor divided in case of (**a**) without Au (**b**) with Au (**c**) with large amount of Au and (**d**) with exceedingly coated Au amount. Reproduced with permission from [88].

More systematic results and analysis of the decoration amount were produced by the work of Wongrat, who used Au nanoparticle decorated ZnO nanostructures for ethanol sensing [88]. They came to several conclusions by conducting the ethanol sensing experiments under different conditions. Sensing results are shown in Table 4. UV illumination activated the room temperature gas sensing property by generating photo-induced oxygen ions that fueled the gas sensing reactions. Au nanoparticles could enhance ZnO sensing response with UV illumination or at elevated temperatures (Figure 60). The sensing mechanisms are illustrated for different sensor designs under different sensing conditions: ZnO based sensor work in dark and with UV illumination, ZnO/Au NPs based sensor work in dark and with UV illumination. Without light illumination, the adsorbed oxygen molecule catches the thermal electrons to form a stable adsorbed oxygen ion which are difficult to desorb and participate in the reaction with gas molecules, thus resulting in no sensing response. UV illumination generated photoelectrons which were caught by surface adsorbed oxygen molecules and formed the photo-oxygen ions. These photo-oxygen ions were loosely bounded to the surface of ZnO. They were energetic and easy to desorb. These photo-oxygen ions reacted with the interacted gas molecules, thus activated and enhanced the sensing response. The effect of light intensity was studied by conducting the measurement under light intensity ranging from 0 mW/cm^2^ to 4.1 mW/cm^2^. The increased light intensity improved sensing response both at room temperature and elevated temperature (125 °C) (Figure 61). Increasing the amount of Au decoration could improve the sensing response initially but too much Au decoration would lead to an opposite outcome (Figure 62). The Au nanoparticles would induce more oxygen ions adsorption by acting as a catalyst. A spill-over region was formed around the AuNPs due to the spill-over effect, where the electrons in ZnO conduction band were easier to be captured by oxygen molecules and enlarged the depletion layer width, thus improve the gas sensing response (Figure 63). As shown in Figure 63c,d, too many Au nanoparticles would coagulate to larger islands and even form a film which would decrease or cover the surface entirely. Consequently, the surface would have fewer sensing reactive sites, hence lower sensing response.

**Figure 64:**
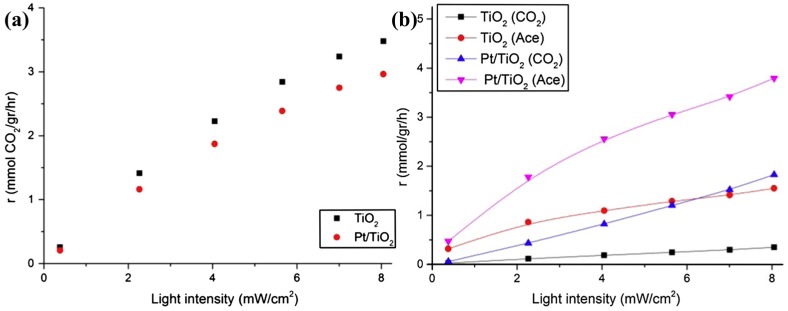
(**a**) CO_2_ production rate (r) versus light intensity for oxidation of propane and (**b**) CO_2_ and acetaldehyde production rate (r) versus light intensities for the photocatalytic oxidation of ethanol on TiO_2_ and Pt-TiO_2_. Reproduced with permission from [89].

The metal decoration could not only improve the sensing response and lowering working temperature. In fact, Fraters reported that it can also enhance selectivity. They investigated the gas-phase photocatalytic oxidation property of TiO_2_ to propane and ethanol with UV illumination. Pt decoration decreased photocatalytic oxidation of propane while it would do the opposite to ethanol (Figure 64). The results were obtained by measuring the production rates of the products of the photocatalytic oxidation process. Though their work was not primarily about gas sensing, but the photocatalytic mechanism was the same. Therefore, their work confirmed that Pt decoration on metal oxides improved sensing selectivity [89].

#### 3.1.3. Summary 

Two kinds of heterostructures have been reviewed: inorganic semiconductor-metal oxides and metal-metal oxides. Heterostructures generally bring improvement in sensing response, sensing speed, reduction of working temperature. Activation of sensing response using visible light, one can even improve selectivity. The effect has been attributed to the facilitation of electron transportation and suppression of recombination, thus resulting in an increase in electron concentration. Heterostructures also improve sensor performance by combining the merits of different materials, for example, some materials might act as a sensitizer to enable visible light activation. Metal has also been found to offer catalytic and spill-over effects. This mechanism was confirmed by resistance, photovoltage, phase measurement and light adsorption spectra. The ratio of the constituent components often required optimization in order to achieve a balance between effective surface area and carrier concentration. Light intensity should also be optimized to reach the balance between photoelectron generation and gas desorption. Heterostructures that combined metal-oxides with carbon-based nanostructures like graphene, carbon nanotubes [90,91,92,93], and organic polymers [94,95] to improve the gas sensing properties have been reported. They require another dedicated review to cover the extensive amount of work published so far, and are therefore not included in this review.

### 3.2. Metal Oxides with Doping

By adding dopant to the metal oxide, it is possible to reduce the resistance baseline of the device and improve sensing performance. Hara reported that both donor (Nb^5+^) and acceptor (Fe^3+^) could decrease the resistance of SrTiO_3_ which is highly sensitive towards O_2_ at room temperature, but resulted in significant lost in sensing performance. Only acceptor (Cr^3+^) was good for both decreasing the resistance for measurement and maintaining the gas sensing property of the material [96].

**Figure 65:**
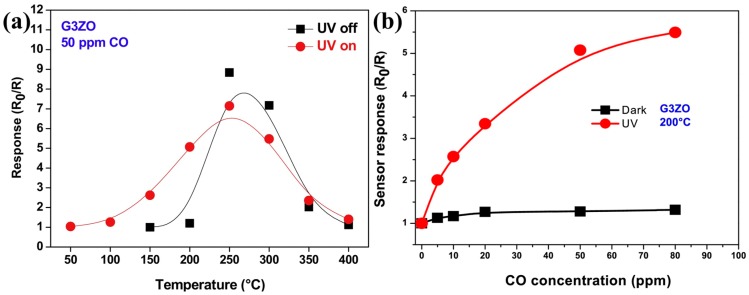
CO sensing response of the Ga doped ZnO (G3ZO) sensor with and without UV light irradiation (**a**) at different temperatures; (**b**) at 200 °C towards different concentrations. Reproduced with permission from [97].

**Figure 66:**
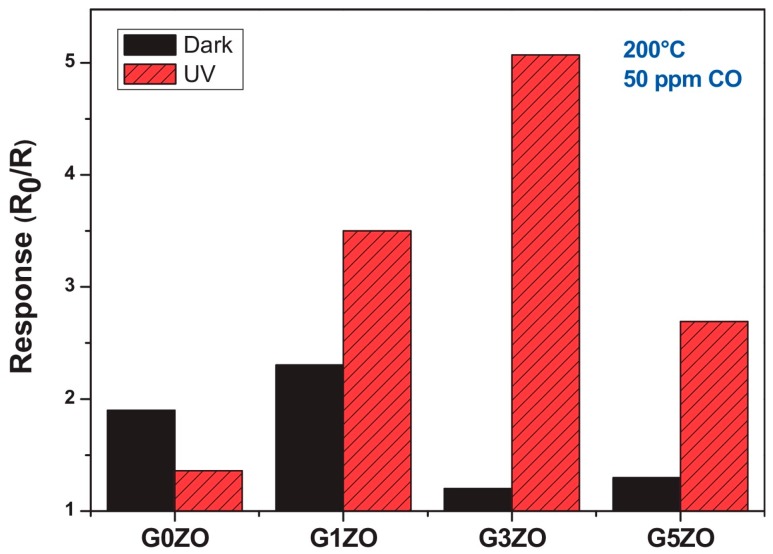
CO sensing response of pure ZnO and ZnO doped with Ga at different concentrations in dark and under UV illumination. Reproduced with permission from [97].

Dhahri doped ZnO nanoflowers with Ga of different amount and used the material to detect CO at different temperatures. UV irradiation would decrease the resistance baseline, lower the optimal sensors working temperature, increase CO sensing response and accelerate the sensing speed due to the photon generated electrons and photon induced gas desorption (Figure 65). The decrease of green light emission and increase of UV emission in the photoluminescence (PL) spectra indicated that Ga atoms occupied Zn vacancy defects. Ga doped ZnO exhibited lower resistance baseline and higher CO sensing response than pristine ZnO both in dark and under UV light illumination. The response was also dependent on the amount of Ga doping. Increasing Ga amount led to an increase in sensing response at first but afterwards decreased the response (Figure 66). This was explained by the fact that Ga doping caused Ga^3+^ to occupy Zn^2+^ vacancies and released electrons and provide more oxygen adsorption active sites [97].

**Figure 67:**
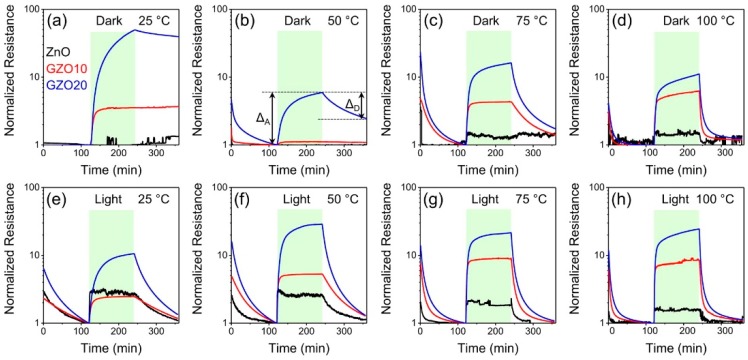
Normalized resistance changes to 400 ppb NO_2_ (green shaded box) for ZnO and ZnO with various amount Ga doping (GZO10, GZO20) in dark (**a**–**d**) and with 460 nm visible light illumination (**e**–**h**) at temperatures ranging from 25 °C to 100 °C. Reproduced with permission from [98].

Sturaro used the same materials: gallium doped ZnO nanocrystals for sensing of reducing H_2_ and oxidizing NO_2_. They compared the sensing property of ZnO doped with different amount of Ga at different operation temperatures in dark and with 460 nm visible light irradiation. As shown in Figure 67, Ga doping improved the sensing response to NO_2_ at temperatures ranging from 25 °C to 100 °C in both dark and with purple-blue light irradiation. The improvement in sensing response by Ga doping was attributed to the released electrons and increased oxygen active adsorption sites [98].

**Figure 68:**
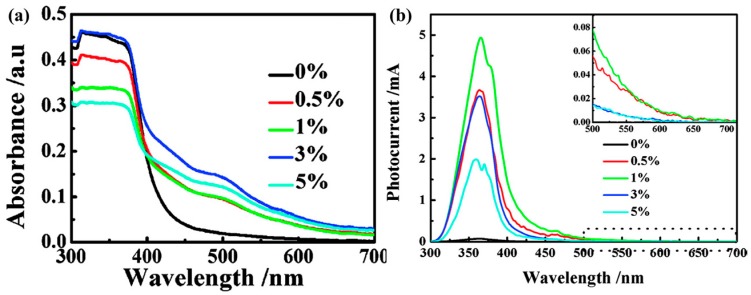
(**a**) Absorption spectrum and (**b**) Photocurrent of Fe-doped flowerlike ZnO with the ratio of Fe/ZnO from 0.1 mol % to 5 mol %. Reproduced with permission from [99].

**Figure 69:**
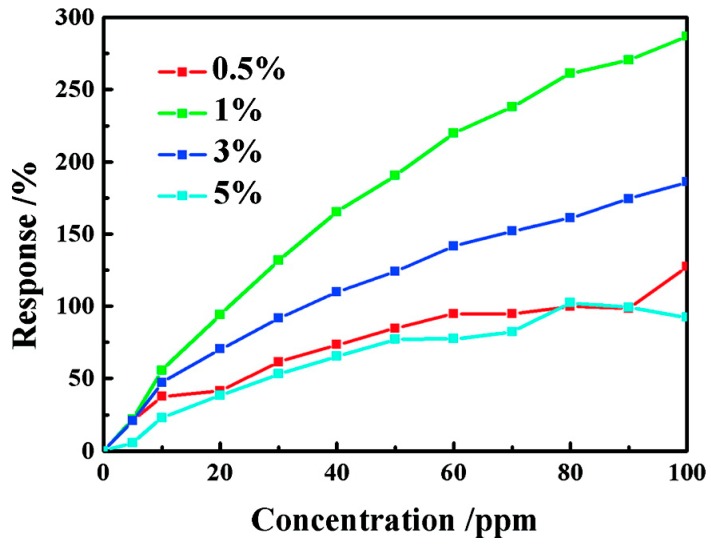
HCHO sensing response of Fe-doped ZnO nanoflowers (with the ratio from 0.5 mol % to 5 mol %) under 532 nm light illumination. Reproduced with permission from [99].

Han synthesized Fe-doped ZnO flowers for HCHO sensing at room temperature. XRD results and UV-vis spectra confirmed the successful doping of Fe ions into ZnO crystal lattice. The UV-vis absorption spectra and photocurrent of Fe-doped ZnO revealed that the absorption spectra were extended to the visible range (Figure 68). Room temperature HCHO sensing was activated by 532 nm light and the sensing response among materials with different amount of Fe doping was compared. As shown in Figure 69, increasing Fe doping resulted in increasing sensing response. However, too much Fe doping would then decrease HCHO sensing because too much Fe dopant would result in the formation of a second phase, i.e., ZnFe_2_O_4_ which resulted in high density of grain boundaries that would inhibit carrier transporting. Besides, ZnFe_2_O_4_ would occupy the active sites on the surface of ZnO thus reduced the chemical absorption of oxygen ions [99].

After two years, they published another paper about the HCHO sensing property of Fe-doped ZnO under visible light illumination. Besides the doping concentration, annealing temperature influenced the sensing property as well. Annealing the Fe-doped ZnO at high temperatures induced visible light absorption which is shown by the absorbance spectra and photocurrent results. The HCHO sensing is activated by 532 nm light. By increasing the annealing temperature, the photocurrent and HCHO sensing response increased and exhibited the highest response at 600 °C. The improvement is attributed to improved crystal quality and increased active sites on the surface of ZnO. However, the response to light and HCHO decreased when the sensor was annealed at a temperature higher than 700 °C. This was because the annealing temperature influenced the position of Fe atoms in ZnO. Between 400 °C and 600 °C, Fe existed in ZnO lattice as ions while after 700 °C, Fe formed ZnFe_2_O_4_ which occupied the active sites and increased grain boundaries that inhibited carriers transport [100].

**Figure 70:**
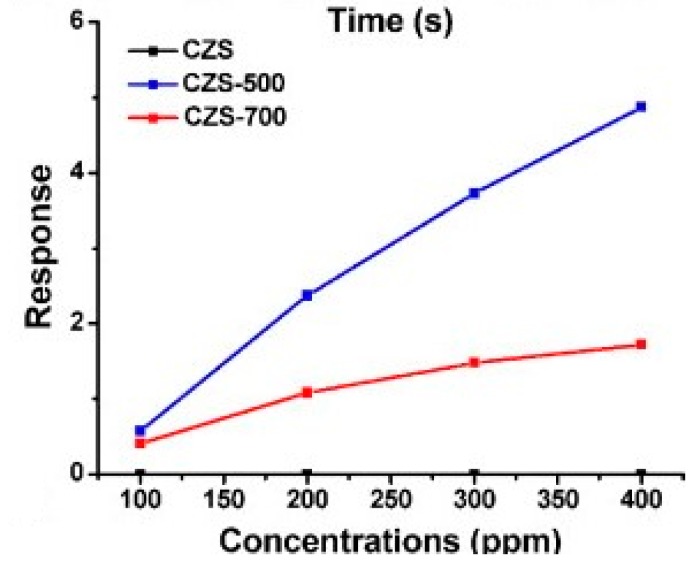
Ethanol sensing response of C doped ZnO microspheres with different calcination temperatures. Reproduced with permission from [101].

This annealing temperature dependence was also observed by Zhai who synthesized carbon-doped ZnO microspheres to detect ethanol vapors at room temperature with UV illumination. The carbon doped ZnO microspheres were annealed at 500 °C and 700 °C. The 500 °C annealed sensor exhibited the highest ethanol sensing response compared to 700 °C annealed samples and non-annealed samples (Figure 70). The transient photovoltage (TPV) measurements and Raman spectroscopy confirmed that Sp^2^ carbon-type structures were formed after the 500 °C annealing and these structures improved carrier separation and restrained the recombination of photon generated carriers [101].

#### Summary

This part summarizes the reported results of light-activated gas sensors with the incorporation of dopant, which decreased the resistance baseline, improved sensing response and enabled visible light activation. Detailed summary of these reported work is illuminated in Table 5. Choice of dopant material, concentration and sensors calcination temperature significantly influenced the sensing outcome. It was possible to find an optimal doping amount and annealing temperature for achieving best performance through obtaining the correct crystal property and increasing the density of active sites. However, by now research on the light-activated sensors based on metal oxides with doping is far from sufficient. More research effort could be conducted.

## 4. Challenges and Outlook

Using photons to activate conductometric gas sensor enables room temperature gas detection. Compared to their conventional thermal energy activated counterparts, the main disadvantage of light-activated sensors is that the sensing response might be much lower, which in turn translates into weak sensitivity limit. Hien demonstrated that SnO_2_ exhibited sensing performance towards H_2_S with high stability at room temperature under violet illumination, the response was only 1.25 to 20 ppm of H_2_S [102]. Zheng used ZnO nanoparticles for the sensing material, and their device exhibited a response of 1.3 to 200 ppm of ethanol vapor under 5 mW of 370 nm illumination at room temperature, whereas no response was observed after removing the light illumination [103]. In fact, previous light-activated room-temperature chemi-resistive gas sensors usually also exhibited low response [10,37,102,103,104]. Lin reported the use of ZnO nanowires for sensing ethanol and their device showed a response of 1.25, i.e., the ratio between initial resistance (R_a_) and resistance with the presence of target gas (R_g_), with 150 ppm of ethanol under the illumination of AM 1.5 G (Standard solar spectral irradiance at air mass 1.5: Direct normal and hemispherical for a 37 degree tilted surface) at 53 °C [104]. Geng used a 4 W white light LED to activate ZnO for detecting acetone at room temperature and the response was only 1.2 for 900 ppm acetone [37]. More published data on gas sensing response based on the light-activated conductometric gas sensor are summarized in Table 6. The data clearly reveals the low response towards different gas types with concentrations as high as 1000 ppm. Generally, the sensing responses are below 2.

Since the sensing response is highly dependent on electron concentration, light absorption efficiency plays a crucial role. Likewise, the operation of solar cells, photocatalysis, light emission devices and photodetectors are dependent on the concentration of photo-generated electrons. A large number of research efforts have been undertaken for these systems to take advantage of the concept of localized surface plasmon resonance (LSPR) to improve device performance [107,108,109,110,111,112,113,114,115,116,117]. LSPR is associated with the oscillation of electrons in noble metal nanoparticles stimulated by an incident light with matched wavelength. LSPR holds a strong capacity for enhancing charge generation and separation through resonant energy transfer across the metal-semiconductor. LSPR improve optoelectronic properties by expanding the light absorption spectrum range and increasing photo-electrons concentration [118,119,120,121]. Four main effects induced by LSPR are usually used to explain the improved properties:

(1) LSPR absorption of noble metal nanoparticles extends the absorption spectrum to the visible range;
(2) Hot electrons emerged in noble metal nanoparticles may result in carrier injection into the conduction band of the semiconductor, hence leading to an increase in the concentration of energetic electrons;
(3) Schottky barrier formed at the interface between the noble metal and metal oxides can suppress recombination of electron-hole pairs;
(4) Localized electric filed enhancement can increase the separation of election-hole pairs on the semiconductor side.

**Figure 71:**
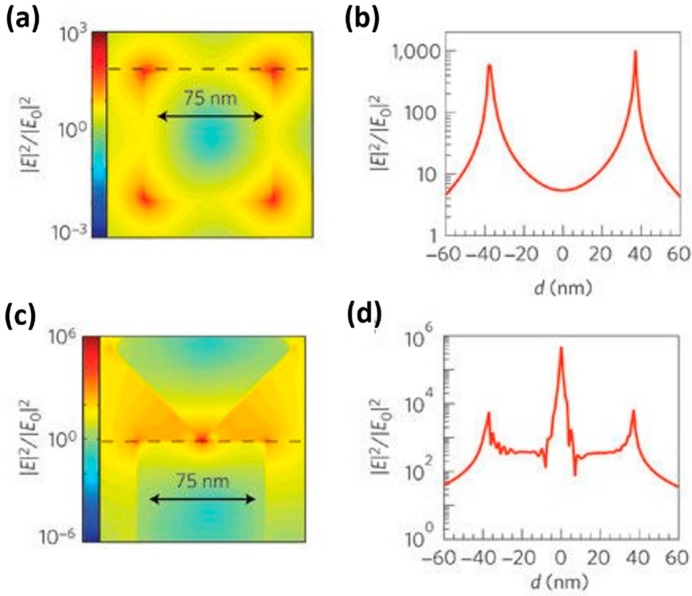
Finite difference time domain (FDTD) simulation of electric field intensity spatial distribution under 420 nm irradiation of (**a**) single 75 nm Ag nanocube; (**c**) two 75 nm Ag nanocubes separated by a distance of 1 nm (one cube is rotated 45°); (**b**,**d**) Electric field intensity enhancement as a function of distance along the dashed line in left figure respectively. Reproduced with permission from [122].

**Figure 72:**
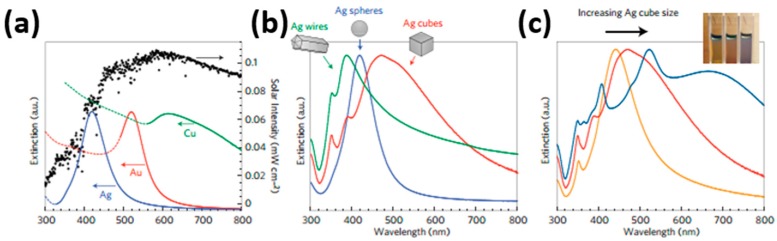
Normalized extinction spectra of (**a**) spherical Ag, Au, and Cu; (**b**) Ag nanoparticles with different nanostructure: wires, spheres, cubes; (**c**) Ag nanocubes as a function of size. The inset in (**c**) is an image of the Ag nanocubes of different sizes suspended in ethanol respectively. Reproduced with permission from [122].

**Figure 73:**
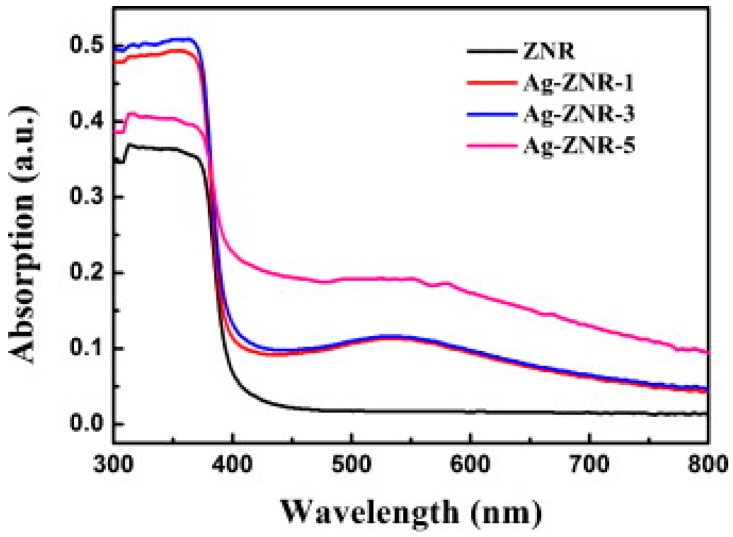
Absorption spectrum of pure ZnO nanorods and ZnO nanorods decorated with Ag nanoparticles of different amount. Reproduced with permission from [86].

**Figure 74:**
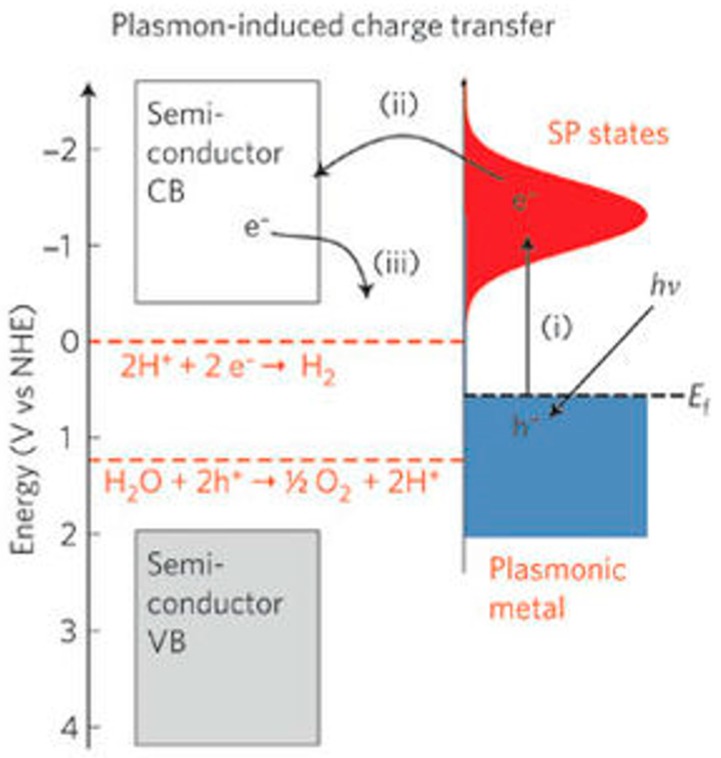
LSPR induced charge transfer with approximate energy level on the normal hydrogen electrode (NHE) scale. Dashed red lines refer to the water-splitting redox potentials. Reproduced with permission from [122].

Linic reviewed the progress in plasmon-assisted photocatalysis, which is based on semiconductor nanostructures decorated with noble metal. They analyzed the improvement induced by LSPR for water splitting. They used finite difference time domain (FDTD) simulations to study the electric field distribution in and around Ag nanocubes under 420 nm light irradiation. Due to the strong concentration of photon flux within small volumes, the field intensity increased by nearly 1000 times around a single Ag nanocube. The localized field enhancement even reached 10^6^ times in the region sandwiched between two Ag nanocubes (Figure 71). The enhanced electric field can facilitate separation of electron-hole pairs in semiconductors significantly, and thus leading to increase in carrier generation [122]. Moreover, the metal nanoparticles exhibited visible light absorption due to their surface plasmon resonance, which provided the merit of visible light activation. The adsorption spectrum could be tuned by controlling their shape and size (Figure 72). Similar expansion of the light absorption spectrum was also observed by Cui, who studied the HCHO sensing of Ag-ZnO nanorod (Figure 73) [86]. Again, the hot electron injection mechanism was used for explaining the LSPR enhancement of optoelectronic properties. Here, they used photocatalytic water splitting as an example to illustrate the hot electron injection model (Figure 74). Upon photons injection, the electrons around the Fermi level of the metal nanoparticles are excited to the surface plasmon resonance state. These energetic electrons are transferred to the conduction band of nearby semiconductor reactions.

As mentioned above, the formation of Schottky barriers could help to separate electron-hole pairs and suppress their recombination. Zhang used a schematic to compare the photocatalysis property of pure TiO_2_ and Au nanoparticles decorated TiO_2_. At the interface between the Au nanoparticle and the TiO_2_ nanoparticle, a space charge region is formed, which induces an internal electric field. This space charge region is the Schottky barriers and the internal electric field forced electron separation and transportation, thus suppressed recombination of electron-hole pairs which is very severe in pure TiO_2_ nanostructures [123].

Consequently, using resonant plasmonic effects to improve the sensing property of light-activated gas sensors should be a promising approach. Noble metal decoration is applied to improve the sensing performance because of the increased carrier concentration due to the heterostructure and combination of advantages of different materials, as discussed in the “Heterostructures containing metal and metal oxide semiconductors” section However, there is nearly no research investigated the LSPR improvement of gas sensing property. Therefore, more efforts can be made to fabricate light-activated gas sensors with metal decoration and to study the roles of LSPR for the improved sensing properties.

The use of composite nanostructures can be used to improve the sensing response. Researchers can further incorporate more new materials to form heterostructures within the gas sensor. Since the availability of results from gas sensors based on metal oxides with doping has been very limit so far, much more efforts should be taken.

Apart from sensing response, gas selectivity is also a significant issue to be improved. By using heterostructures, doping, tuning of light wavelength and temperature, one can control or improve the gas selectivity to some extent. However, the actual signal output is far from enough. An optimal gas sensor should only be sensitive to a certain gas. It would be very important to find materials that are only response to a certain gas.

Although light activation has already improved the device long-term stability as compared to thermal-activated device, the situation can be further improved.

Numerous publications have suggested that the sensing mechanism in gas sensor is the surface depletion model. However, there are only a handful of experimental demonstrations on this sensing mechanism. More experimental efforts, including those based on electron paramagnetic resonance (EPR), are needed to study the adsorption species on the surface of the materials. To demonstrate the reactions taking place during the sensing process, the concentrations levels of reactants and products can be monitored by techniques such as Fourier Transform infrared spectroscopy (FTIR), etc.

## 5. Conclusions

In this paper, we have reviewed the progress of light-activated conductometric gas sensors based on metal oxides. We start from the simplest structure, i.e., pure metal oxides. 1D nanostructures and porous nanostructures are discussed specifically due to their high surface-to-volume ratio and large surface area. The size of the nanostructures should also be optimized to make it comparable to the width of the depletion layer. Combining different materials may result in combining their individual merits, which will ultimately benefit over sensing performance. Generally, heterostructures result in higher response and better selectivity due to the increased carrier concentration. Heterostructures can also extend light absorption into the visible range due to the sensitization effect of certain materials. The ratio between different materials in the heterostructure should be optimized for achieving a balance between surface active adsorption sites and carrier concentration. There is only limited reported results to provide a comprehensive investigation on how dopants may enhance the sensing properties of metal oxides. Nonetheless, an appropriate amount of doping has been shown to improve sensing response by increasing carrier generation and enabling visible light activation. Light intensity at specific wavelength can change sensing response and even tune the device’s selectivity. Surprisingly, experimental results suggest that a simple increase of light intensity may not necessarily bring better response. In fact, one has to choose the correct intensity level at some wavelength range for achieving the best performance. There exists a competition between photon generated electrons and photon induced desorption. Film thickness also affects sensing performance because of limited penetration depth of the photons. All these findings are highly instructive in the design and implementation of light-activated gas sensors for optimized performance. In the last part, we point out the main drawbacks of light-activated gas sensors, particularly in relation to the issue of low response. We have also analyzed the feasibility of using LSPR to resolve this problem. This speculative comment may open up a new window for developing room temperature operated gas sensors with sufficiently high response for practical devices.

## Figures and Tables

**Table 1:** Resistance shift in semiconductor material in relation to redox type of target gas.

Type of Semiconducting Material	Reductive Gas	Oxidizing Gas
*n*-type	Resistance decrease	Resistance increase
*p*-type	Resistance increase	Resistance decrease

**Table 2:** Definition of sensor response.

Type of Semiconductor Material	Reductive Gas	Oxidizing Gas
*n*-type	R_a_/R_g_ or (R_a_ − R_g_)/R_g_	R_g_/R_a_ or (R_g_ − R_a_)/R_a_
*p*-type	R_g_/R_a_ or (R_g_ − R_a_)/R_a_	R_a_/R_g_ or (R_a_ − R_g_)/R_g_

**Table 3:** Summary of key reported work on light-activated gas sensor based on metal oxides nanoparticles.

Material	Light Illumination	Temperature	Vapor	Ref
ZnO thin film	ultravlolet (UV)	room-temperature (RT)	H_2_	[36]
ZnO nanoline	UV	RT	H_2_	[36]
ZnO nanoparticle	UV	RT	hexane, propane, methane, ethanol, toluene, acetadyde, acetone and pentane	[38]
ZnO	white LED lamp and visible light with different wavelength	RT	acetone and ethylene	[37]
ZnO nanospheres	white LED	RT	H_2_O_2_	[42]
ZnO thin film	light of different wavelength	RT	NO_2_, methanol, ethanol butanol, ammonia, sufur dioxide, methane, propane, etc.	[9]
ordered porous ZnO arrays	UV	RT	NO_2_	[43]
SnO_2_	UV LED	RT	O_3_	[48]
SnO_2_ film	UV of Hg lamp	RT	H_2_	[47]
SnO_2_	monochromatic light	RT	CO, Ethyl Alcohol, NO_2_	[46]
SnO_2_ pyrolytic films	UV irradiation with different wavelength	RT	acetone and trichloroethylene	[49]
SnO_2_	halogen lamp illumination	RT	NO_2_	[16]
TiO_2_	UV	various temperatures	formaldehyde	[51]
TiO_2_	UV lamps	not available	CO and H_2_	[52]
WO_3_	visible light	RT	NO_2_	[56]
ultrathin layers of In_2_O_3_ nanoparticles	GaInN/GaN based LED	RT	O_3_	[57]
7 nm In_2_O_3_ nanoparticle and 12 nm nanoparticle	RT	RT	O_3_	[58]

**Table 4:** Ethanol sensing results of pure ZnO and Au NPs decorated ZnO under different mearsurement conditions.

**Room Temperature**		
**Material Condition**	**ZnO**	**ZnO:Au NPs**
Dark	No response	No response
UV	Low response	High response
**High Temperature (125 °C)**		
**Material Condition**	**ZnO**	**ZnO:Au NPs**
dark	No response	Have response
UV	Have response	High response

**Table 5:** Summary of typical advances on light-activated gas sensor based on metal oxides with doping.

Material	Light Illumination	Temperature ****	Vapor	Ref
Nb^5+^-doped SrTiO_3_	UV	RT	O_2_	[96]
Fe^3+^-doped SrTiO_3_	UV	RT	O_2_	[96]
Cr^3+^-doped SrTiO_3_	UV	RT	O_2_	[96]
Ga-doped ZnO nanopowder	UV	various temperatures	CO	[97]
Ga-doped ZnO nanocrystal	460 nm visible light	temperatures ranging from 25 °C to 100 °C	H_2_, NO_2_	[98]
Fe-doped ZnO flowers	532 nm light	RT	HCHO	[99]
Fe-doped ZnO flowers	532 nm light	RT	HCHO	[100]
carbon-doped ZnO microspheres	UV	RT	ethanol	[101]

**Table 6:** Summary of response of light-activated sensors operated at room temperature. (The response is defined as the ratio between R_a_ and R_g_: R_a_/R_g_ if there is no special definition in the table. ΔR, ΔG, ΔI is the absolute value of change of resistance, conductance and current respectively).

Material	Light Illumination	Temperature	Vapor	Concentration (ppm)	Response	Ref
ZnO	UV 3.6 mW/cm^2^ (365 nm)	RT	Ethanol	100	0.14	[64]
SnO_2_-GaN nanowires	UV 3.75 mW/m^2^ (365 nm)	RT	Ethanol	500	1.01	[105]
ZnO nanodisk	UV 1.6 mW/cm^2^ (365 nm)	RT	Ethanol	100	1.17	[106]
ZnO thin film	UV	RT	H_2_	100	0.1 ΔR/R_air_	[36]
ZnO nanoline	UV	RT	H_2_	100	0.2	[36]
ZnO	White light LED	RT	Ethylene	5200	1.06	[37]
ZnO	White light LED	RT	H_2_O_2_	100	0.5 ΔR/R_g_	[42]
SnO_2_	UV	RT	CO	200	0.84 ΔG/G_air_	[16]
TiO_2_ NWs-80 nm	UV	RT	NH_3_	100	0.5 ΔZ/Z_air_	[60]
TiO_2_ NWs-550 nm	UV	RT	NH_3_	100	0.1	[60]
ZnO	UV	RT	Ethanol	60	0.8 ΔI/I_air_	[14]
ZnO	Visible light	RT	HCHO	110	1.35% ΔI/I_air_	[10]
Cu/ZnO	355 nm	RT	ethanol	1120	64% ΔI/I_air_	[11]
Cu/ZnO	355 nm	RT	acetone	1120	63% ΔI/I_air_	[11]

## References

[1] Volanti D.P., Felix A.A., Orlandi M.O., Whitfield G., Yang D.J., Longo E., Tuller H.L., Varela J.A. (2013). The role of hierarchical morphologies in the superior gas sensing performance of CuO-based chemiresistors. Adv. Funct. Mater..

[2] Shin J., Choi S.J., Lee I., Youn D.Y., Park C.O., Lee J.H., Tuller H.L., Kim I.D. (2013). Thin-wall assembled SnO_2_ fibers functionalized by catalytic Pt nanoparticles and their superior exhaled-breath-sensing properties for the diagnosis of diabetes. Adv. Funct. Mater..

[3] Katwal G., Paulose M., Rusakova I.A., Martinez J.E., Varghese O.K. (2016). Rapid growth of zinc oxide nanotube-nanowire hybrid architectures and their use in breast cancer-related volatile organics detection. Nano Lett..

[4] Kim S.J., Choi S.J., Jang J.S., Kim N.H., Hakim M., Tuller H.L., Kim I.D. (2016). Mesoporous WO_3_ nanofibers with protein-templated nanoscale catalysts for detection of trace biomarkers in exhaled breath. ACS Nano.

[5] Andringa A.M., Piliego C., Katsouras I., Blom P.W., de Leeuw D.M. (2014). NO_2_ detection and real-time sensing with field-effect transistors. Chem. Mat..

[6] Hagedorn K., Li W., Liang Q., Dilger S., Noebels M., Wagner M.R., Reparaz J.S., Dollinger A., auf der Günne J.S., Dekorsy T. (2016). Catalytically doped semiconductors for chemical gas sensing: aerogel-like aluminum-containing zinc oxide materials prepared in the gas phase. Adv. Funct. Mater..

[7] Dong X., Li T., Liu Y., Li Y., Zhao C.L., Chan C.C. (2011). Polyvinyl alcohol-coated hybrid fiber grating for relative humidity sensing. J. Biomed. Opt..

[8] Zhang S., Dong X., Li T., Chan C.C., Shum P.P. (2013). Simultaneous measurement of relative humidity and temperature with PCF-MZI cascaded by fiber Bragg grating. Opt. Commun..

[9] Fabbri B., Gaiardo A., Giberti A., Guidi V., Malagù C., Martucci A., Sturaro M., Zonta G., Gherardi S., Bernardoni P. (2016). Chemoresistive properties of photo-activated thin and thick ZnO films. Sens. Actuator B.

[10] Zhai J., Wang D., Peng L., Lin Y., Li X., Xie T. (2010). Visible-light-induced photoelectric gas sensing to formaldehyde based on CdS nanoparticles/ZnO heterostructures. Sens. Actuator B.

[11] Peng L., Xie T.-F., Yang M., Wang P., Xu D., Pang S., Wang D.-J. (2008). Light induced enhancing gas sensitivity of copper-doped zinc oxide at room temperature. Sens. Actuator B.

[12] Şahin Y., Öztürk S., Kılınç N., Kösemen A., Erkovan M., Öztürk Z.Z. (2014). Electrical conduction and NO_2_ gas sensing properties of ZnO nanorods. Appl. Surf. Sci..

[13] Peng L., Zhai J., Wang D., Zhang Y., Wang P., Zhao Q., Xie T. (2010). Size- and photoelectric characteristics-dependent formaldehyde sensitivity of ZnO irradiated with UV light. Sens. Actuator B.

[14] Gong J., Li Y., Chai X., Hu Z., Deng Y. (2009). UV-light-activated ZnO fibers for organic gas sensing at room temperature. J. Phys. Chem. C.

[15] Chen Y., Li X., Li X., Wang J., Tang Z. (2016). UV activated hollow ZnO microspheres for selective ethanol sensors at low temperatures. Sens. Actuator B.

[16] Comini E., Cristalli A., Faglia G., Sberveglieri G. (2000). Light enhanced gas sensing properties of indium oxide and tin dioxide sensors. Sens. Actuator B.

[17] Wang F., Li H., Yuan Z., Sun Y., Chang F., Deng H., Xie L., Li H. (2016). A highly sensitive gas sensor based on CuO nanoparticles synthetized via a sol–gel method. RSC Adv..

[18] Liu M., Ren S.P., Zhang R.Y., Xue Z.Y., Ma C.R., Yin M.L., Xu X., Bao S.Y., Chen C.L. (2015). Gas sensing properties of epitaxial LaBaCo2O5.5+delta thin films. Sci. Rep..

[19] Zou A.L., Qiu Y., Yu J.J., Yin B., Cao G.Y., Zhang H.Q., Hu L.Z. (2016). Ethanol sensing with Au-modified ZnO microwires. Sens. Actuator B.

[20] Li Z., Zhou Y., Song J., Yu T., Liu J., Zou Z. (2013). Versatile nanobead-scaffolded N-SnO_2_ mesoporous microspheres: One-step synthesis and superb performance in dye-sensitized solar cell, gas sensor, and photocatalytic degradation of dye. J. Mater. Chem. A.

[21] Saboor F.H., Ueda T., Kamada K., Hyodo T., Mortazavi Y., Khodadadi A.A., Shimizu Y. (2016). Enhanced NO_2_ gas sensing performance of bare and Pd-loaded SnO_2_ thick film sensors under UV-light irradiation at room temperature. Sens. Actuator B.

[22] Barsan N., Weimar U. (2001). Conduction model of metal oxide gas sensors. J. Electroceram..

[23] Yamazoe N., Sakai G., Shimanoe K. (2003). Oxide semiconductor gas sensors. Catal. Surv. Asia.

[24] Zhang J., Liu X., Neri G., Pinna N. (2016). Nanostructured materials for room-temperature gas sensors. Adv. Mater..

[25] Comini E., Baratto C., Faglia G., Ferroni M., Vomiero A., Sberveglieri G. (2009). Quasi-one dimensional metal oxide semiconductors: Preparation, characterization and application as chemical sensors. Prog. Mater. Sci..

[26] Lee J.-H. (2009). Gas sensors using hierarchical and hollow oxide nanostructures: Overview. Sens. Actuator B.

[27] Tiemann M. (2007). Porous metal oxides as gas sensors. Chem. Eur. J..

[28] Franke M.E., Koplin T.J., Simon U. (2006). Metal and metal oxide nanoparticles in chemiresistors: Does the nanoscale matter?. Small.

[29] Miller D.R., Akbar S.A., Morris P.A. (2014). Nanoscale metal oxide-based heterojunctions for gas sensing: A review. Sens. Actuator B.

[30] Smulko J.M., Trawka M., Granqvist C.G., Ionescu R., Annanouch F., Llobet E., Kish L.B. (2015). New approaches for improving selectivity and sensitivity of resistive gas sensors: A review. Sens. Rev..

[31] Arya S.K., Krishnan S., Silva H., Jean S., Bhansali S. (2012). Advances in materials for room temperature hydrogen sensors. Analyst.

[32] Lin Y., Kan K., Song W., Zhang G., Dang L., Xie Y., Shen P., Li L., Shi K. (2015). Controllable synthesis of Co_3_O_4_/polyethyleneimine-carbon nanotubes nanocomposites for CO and NH_3_ gas sensing at room temperature. J. Alloy. Compd..

[33] Liu S., Zhou L., Yao L., Chai L., Li L., Zhang G., Shi K. (2014). One-pot reflux method synthesis of cobalt hydroxide nanoflake-reduced graphene oxide hybrid and their NOx gas sensors at room temperature. J. Alloy Compd..

[34] Zhang M., Zhen Y., Sun F., Xu C. (2016). Hydrothermally synthesized SnO_2_-graphene composites for H_2_ sensing at low operating temperature. Mater. Sci. Eng. B.

[35] Seiyama T., Kato A., Fujiishi K., Nagatani M. (1962). A new detector for gaseous components using semiconductive thin films. Anal. Chem..

[36] Fan S.W., Srivastava A.K., Dravid V.P. (2009). UV-activated room-temperature gas sensing mechanism of polycrystalline ZnO. Appl. Phys. Lett..

[37] Geng Q., He Z., Chen X., Dai W., Wang X. (2013). Gas sensing property of ZnO under visible light irradiation at room temperature. Sens. Actuator B.

[38] De Lacy Costello B.P., Ewen R.J., Ratcliffe N.M., Richards M. (2008). Highly sensitive room temperature sensors based on the UV-LED activation of zinc oxide nanoparticles. Sens. Actuator B.

[39] Prades J.D., Jimenez-Diaz R., Hernandez-Ramirez F., Barth S., Cirera A., Romano-Rodriguez A., Mathur S., Morante J.R. (2009). Equivalence between thermal and room temperature UV light-modulated responses of gas sensors based on individual SnO_2_ nanowires. Sens. Actuator B.

[40] Prades J.D., Jimenez-Diaz R., Manzanares M., Hernandez-Ramirez F., Andreu T., Cirera A., Romano-Rodriguez A., Morantea J.R. (2009). Photoexcited individual nanowires: key elements in room temperature detection of oxidizing gases. AIP Conf. Proc..

[41] Yang Z., Guo L., Zu B., Guo Y., Xu T., Dou X. (2014). CdS/ZnO core/shell nanowire-built films for enhanced photodetecting and optoelectronic gas-sensing applications. Adv. Opt. Mater..

[42] Parthasarathy S., Nandhini V., Jeyaprakash B.G. (2016). Improved sensing response of photo activated ZnO thin film for hydrogen peroxide detection. J. Colloid Interface Sci..

[43] Su X., Duan G., Xu Z., Zhou F., Cai W. (2017). Structure and thickness-dependent gas sensing responses to NO_2_ under UV irradiation for the multilayered ZnO micro/nanostructured porous thin films. J. Colloid Interface Sci..

[44] Procek M., Pustelny T., Stolarczyk A. (2016). Influence of External Gaseous Environments on the Electrical Properties of ZnO Nanostructures Obtained by a Hydrothermal Method. Nanomaterials.

[45] Yu C.-C., Hsu Y.-T., Lan W.-H., Shih M.-C., Hong J.-H., Huang K.-F., Huang C.-J. (2013). UV enhanced oxygen response resistance ratio of ZnO prepared by thermally oxidized zn on sapphire substrate. J. Nanomater..

[46] Faglia G., Baratto C., Comini E., Sberveglieri G. (2002). A selective semiconductor gas sensor based on surface photovoltage. Proc. SPIE.

[47] Ao D., Ichimura M. (2012). UV irradiation effects on hydrogen sensors based on SnO_2_ thin films fabricated by the photochemical deposition. Solid-State Electron..

[48] Jeng C.C., Chong P.J.H., Chiu C.C., Jiang G.J., Lin H.J., Wu R.J., Wu C.H. (2014). A dynamic equilibrium method for the SnO_2_-based ozone sensors using UV-LED continuous irradiation. Sens. Actuator B.

[49] Saura J. (1994). Gas-sensing properties of SnO_2_ pyrolytic films subjected toultraviolet radiation. Sens. Actuator B.

[50] Anothainart K., Burgmair M., Karthigeyan A., Zimmer M., Eisele I. (2003). Light enhanced NO_2_ gas sensing with tin oxide at room temperature: Conductance and work function measurements. Sens. Actuator B.

[51] Zhang S., Lei T., Li D., Zhang G., Xie C. (2014). UV light activation of TiO_2_ for sensing formaldehyde: How to be sensitive, recovering fast, and humidity less sensitive. Sens. Actuator B.

[52] Peng X., He Z., Yang K., Chen X., Wang X., Dai W., Fu X. (2016). Correlation between donating or accepting electron behavior of the adsorbed CO or H_2_ and its oxidation over TiO_2_ under ultraviolet light irradiation. Appl. Surf. Sci..

[53] Wang C.Y., Kinzer M., Youn S.K., Ramgir N., Kunzer M., Köhler K., Zacharias M., Cimalla V. (2011). Oxidation behaviour of carbon monoxide at the photostimulated surface of ZnO nanowires. J. Phys. D.

[54] Trawka M.P., Smulko J.M., Hasse L.Z., Granqvist C.G., Ionescu R., Llobet E., Annanouch F.E., Kish L.B. (2016). UV-light-induced fluctuation enhanced sensing by WO_3_-based gas sensors. IEEE Sens. J..

[55] Trawka M., Smulko J., Hasse L., Granqvist C.G., Annanouch F.E., Ionescu R. (2016). Fluctuation enhanced gas sensing with WO_3_-based nanoparticle gas sensors modulated by UV light at selected wavelengths. Sens. Actuator B.

[56] Zhang C., Boudiba A., de Marco P., Snyders R., Olivier M.G., Debliquy M. (2013). Room temperature responses of visible-light illuminated WO_3_ sensors to NO_2_ in sub-ppm range. Sens. Actuator B.

[57] Wang C.Y., Cimalla V., Kups T., Röhlig C.C., Stauden T., Ambacher O., Kunzer M., Passow T., Schirmacher W., Pletschen W. (2007). Integration of In_2_O_3_ nanoparticle based ozone sensors with GaInN/GaN light emitting diodes. Appl. Phys. Lett..

[58] Wang C.Y., Becker R.W., Passow T., Pletschen W., Köhler K., Cimalla V., Ambacher O. (2011). Photon stimulated sensor based on indium oxide nanoparticles I: Wide-concentration-range ozone monitoring in air. Sens. Actuator B.

[59] Kiasari N.M., Soltanian S., Gholamkhass B., Servati P. (2014). Environmental gas and light sensing using ZnO nanowires. IEEE Trans. Nanotechnol..

[60] Wang S., Lin Z.X., Wang W.H., Kuo C.L., Hwang K.C., Hong C.C. (2014). Self-regenerating photocatalytic sensor based on dielectrophoretically assembled TiO_2_ nanowires for chemical vapor sensing. Sens. Actuator B.

[61] Hansen B.J., Kouklin N., Lu G., Lin I.K., Chen J., Zhang X. (2010). Transport, analyte detection, and opto-electronic response of p-type CuO nanowires. J. Phys. Chem. C.

[62] Zampetti E., Macagnano A., Bearzotti A. (2013). Gas sensor based on photoconductive electrospun titania nanofibres operating at room temperature. J. Nanopart. Res..

[63] Su X., Gao L., Zhou F., Cai W., Duan G. (2017). “Close network” effect of a ZnO micro/nanoporous array allows high UV-irradiated NO_2_ sensing performance. RSC Adv..

[64] Chen H., Liu Y., Xie C., Wu J., Zeng D., Liao Y. (2012). A comparative study on UV light activated porous TiO_2_ and ZnO film sensors for gas sensing at room temperature. Ceram. Int..

[65] Liu L., Li X., Dutta P.K., Wang J. (2013). Room temperature impedance spectroscopy-based sensing of formaldehyde with porous TiO_2_ under UV illumination. Sens. Actuator B.

[66] Li X., Chen N., Lin S., Wang J., Zhang J. (2015). NiO-wrapped mesoporous TiO_2_ microspheres based selective ammonia sensor at room temperature. Sens. Actuator B.

[67] Wagner T., Kohl C.D., Morandi S., Malagu C., Donato N., Latino M., Neri G., Tiemann M. (2012). Photoreduction of mesoporous In_2_O_3_: Mechanistic model and utility in gas sensing. Chem. Eur. J..

[68] Wagner T., Kohl C.D., Malagù C., Donato N., Latino M., Neri G., Tiemann M. (2013). UV light-enhanced NO_2_ sensing by mesoporous In_2_O_3_: Interpretation of results by a new sensing model. Sens. Actuator B.

[69] Klaus D., Klawinski D., Amrehn S., Tiemann M., Wagner T. (2015). Light-activated resistive ozone sensing at room temperature utilizing nanoporous In_2_O_3_ particles: Influence of particle size. Sens. Actuator B.

[70] Deng L., Ding X., Zeng D., Tian S., Li H., Xie C. (2012). Visible-light activate mesoporous WO_3_ sensors with enhanced formaldehyde-sensing property at room temperature. Sens. Actuator B.

[71] Wu B., Lin Z., Sheng M., Hou S., Xu J. (2016). Visible-light activated ZnO/CdSe heterostructure-based gas sensors with low operating temperature. Appl. Surf. Sci..

[72] Han L., Wang D., Cui J., Chen L., Jiang T., Lin Y. (2012). Study on formaldehyde gas-sensing of In_2_O_3_-sensitized ZnO nanoflowers under visible light irradiation at room temperature. J. Mater. Chem..

[73] Geng X., Zhang C., Debliquy M. (2016). Cadmium sulfide activated zinc oxide coatings deposited by liquid plasma spray for room temperature nitrogen dioxide detection under visible light illumination. Ceram. Int..

[74] Lu G., Xu J., Sun J., Yu Y., Zhang Y., Liu F. (2012). UV-enhanced room temperature NO_2_ sensor using ZnO nanorods modified with SnO_2_ nanoparticles. Sens. Actuator B.

[75] Hoffmann M.W., Gad A.E., Prades J.D., Hernandez-Ramirez F., Fiz R., Shen H., Mathur S. (2013). Solar diode sensor: Sensing mechanism and applications. Nano Energy.

[76] Yang M., Wang D., Peng L., Zhao Q., Lin Y., Wei X. (2006). Surface photocurrent gas sensor with properties dependent on Ru(dcbpy)_2_(NCS)_2_-sensitized ZnO nanoparticles. Sens. Actuator B.

[77] Chizhov A.S., Rumyantseva M.N., Vasiliev R.B., Filatova D.G., Drozdov K.A., Krylov I.V., Abakumov A.M., Gaskov A.M. (2014). Visible light activated room temperature gas sensors based on nanocrystalline ZnO sensitized with CdSe quantum dots. Sens. Actuator B.

[78] Chizhov A.S., Rumyantseva M.N., Vasiliev R.B., Filatova D.G., Drozdov K.A., Krylov I.V., Marchevsky A.V., Karakulina O.M., Abakumov A.M., Gaskov A.M. (2016). Visible light activation of room temperature NO_2_ gas sensors based on ZnO, SnO_2_ and In_2_O_3_ sensitized with CdSe quantum dots. Thin Solid Films.

[79] Park S., Kim S., Ko H., Lee C. (2014). Light Assisted Room Temperature Ethanol Gas Sensing of ZnO–ZnS Nanowires. J. Nanosci. Nanotechnol..

[80] Park S., Ko H., Lee S., Kim H., Lee C. (2014). Light-activated gas sensing of Bi_2_O_3_-core/ZnO-shell nanobelt gas sensors. Thin Solid Films.

[81] Karaduman I., Yıldız D.E., Sincar M.M., Acar S. (2014). UV light activated gas sensor for NO_2_ detection. Mater. Sci. Semicond. Process..

[82] Han C.H., Hong D.W., Han S.D., Gwak J., Singh K.C. (2007). Catalytic combustion type hydrogen gas sensor using TiO_2_ and UV-LED. Sens. Actuator B.

[83] Deng Q., Gao S., Lei T., Ling Y., Zhang S., Xie C. (2017). Temperature & light modulation to enhance the selectivity of Pt-modified zinc oxide gas sensor. Sens. Actuator B.

[84] Comini E., Ottini L., Faglia G., Sberveglieri G. (2004). SnO/sub 2/RGTO UV activation for CO monitoring. IEEE Sens. J..

[85] Chen M.H., Lu C.S., Wu R.J. (2014). Novel Pt/TiO_2_–WO_3_ materials irradiated by visible light used in a photoreductive ozone sensor. J. Taiwan Inst. Chem. Eng..

[86] Cui J., Wang D., Xie T., Lin Y. (2013). Study on photoelectric gas-sensing property and photogenerated carrier behavior of Ag–ZnO at the room temperature. Sens. Actuator B.

[87] Dhahri R., Hjiri M., el Mir L., Bonavita A., Iannazzo D., Latino M., Donato N., Leonardi S.G., Neri G. (2016). Gas sensing properties of Al-doped ZnO for UV-activated CO detection. J. Phys. D.

[88] Wongrat E., Chanlek N., Chueaiarrom C., Samransuksamer B., Hongsith N., Choopun S. (2016). Low temperature ethanol response enhancement of ZnO nanostructures sensor decorated with gold nanoparticles exposed to UV illumination. Sens. Actuator A.

[89] Fraters B.D., Amrollahi R., Mul G. (2015). How Pt nanoparticles affect TiO_2_-induced gas-phase photocatalytic oxidation reactions. J. Catal..

[90] Lee J.S., Kwon O.S., Shin D.H., Jang J. (2013). WO_3_ nanonodule-decorated hybrid carbon nanofibers for NO_2_ gas sensor application. J. Mater. Chem. A.

[91] Yang J., Li R., Huo N., Ma W.L., Lu F., Fan C., Yang S., Wei Z., Li J., Li S.S. (2014). Oxygen-induced abnormal photoelectric behavior of a MoO_3_/graphene heterocomposite. RSC Adv..

[92] Zhao M., Yan L., Zhang X., Xu L., Song Z., Chen P., Dong F., Chu W. (2017). Room temperature NH_3_ detection of Ti/graphene devices promoted by visible light illumination. J. Mater. Chem. C.

[93] Ueda T., Takahashi K., Mitsugi F., Ikegami T. (2009). Preparation of single-walled carbon nanotube/TiO_2_ hybrid atmospheric gas sensor operated at ambient temperature. Diam. Relat. Mater..

[94] Gad A., Hoffmann M.W., Casals O., Mayrhofer L., Fàbrega C., Caccamo L., Hernández-Ramírez F., Mohajerani M.S., Moseler M., Shen H. (2016). Integrated strategy toward self-powering and selectivity tuning of semiconductor gas sensors. ACS Sens..

[95] Zhang C., Wang J., Olivier M.G., Debliquy M. (2015). Room temperature nitrogen dioxide sensors based on N719-dye sensitized amorphous zinc oxide sensors performed under visible-light illumination. Sens. Actuator B.

[96] Hara T., Ishiguro T., Wakiya N., Shinozaki K. (2008). Oxygen Sensing Properties of SrTiO_3_ Thin Films. Jpn. J. Appl. Phys..

[97] Dhahri R., Hjiri M., Mir L.E., Bonavita A., Iannazzo D., Leonardi S.G., Neri G. (2015). CO sensing properties under UV radiation of Ga-doped ZnO nanopowders. Appl. Surf. Sci..

[98] Sturaro M., della Gaspera E., Michieli N., Cantalini C., Emamjomeh S.M., Guglielmi M., Martucci A. (2016). Degenerately doped metal oxide nanocrystals as plasmonic and chemoresistive gas sensors. ACS Appl. Mater. Interfaces.

[99] Han L., Wang D., Lu Y., Jiang T., Liu B., Lin Y. (2011). Visible-light-assisted HCHO gas sensing based on Fe-doped flowerlike ZnO at room temperature. J. Phys. Chem. C.

[100] Han L., Wang D., Lu Y., Jiang T., Chen L., Xie T., Lin Y. (2013). Influence of annealing temperature on the photoelectric gas sensing of Fe-doped ZnO under visible light irradiation. Sens. Actuator B.

[101] Zhai J., Wang L., Wang D., Lin Y., He D., Xie T. (2012). UV-illumination room-temperature gas sensing activity of carbon-doped ZnO microspheres. Sens. Actuator B.

[102] Hien V.X., Heo Y.W. (2016). Effects of violet-, green-, and red-laser illumination on gas-sensing properties of SnO thin film. Sens. Actuator B.

[103] Zheng Z.Q., Yao J.D., Wang B., Yang G.W. (2015). Light-controlling, flexible and transparent ethanol gas sensor based on ZnO nanoparticles for wearable devices. Sci. Rep..

[104] Lin C.H., Chang S.J., Chen W.S., Hsueh T.J. (2016). Transparent ZnO-nanowire-based device for UV light detection and ethanol gas sensing on c-Si solar cell. RSC Adv..

[105] Bajpai R., Motayed A., Davydov A.V., Oleshko V.P., Aluri G.S., Bertness K.A., Rao M.V., Zaghloul M.E. (2012). UV-assisted alcohol sensing using SnO_2_ functionalized GaN nanowire devices. Sens. Actuator B.

[106] Alenezi M.R., Alshammari A.S., Jayawardena K.D., Beliatis M.J., Henley S.J., Silva S.R. (2013). Role of the exposed polar facets in the performance of thermally and UV activated ZnO nanostructured gas sensors. J. Phys. Chem. C.

[107] Liu M., de Arquer F.P., Li Y., Lan X., Kim G.H., Voznyy O., Jagadamma L.K., Abbas A.S., Hoogland S., Lu Z. (2016). Double-sided junctions enable high-performance colloidal-quantum-dot photovoltaics. Adv. Mater..

[108] Bai S., Jin Y., Liang X., Ye Z., Wu Z., Sun B., Ma Z., Tang Z., Wang J., Würfel U., Gao F., Zhang F. (2015). Ethanedithiol treatment of solution-processed ZnO thin films: Controlling the intragap states of electron transporting interlayers for efficient and stable inverted organic photovoltaics. Adv. Energy Mater..

[109] Chen X., Lin P., Yan X., Bai Z., Yuan H., Shen Y., Liu Y., Zhang G., Zhang Z., Zhang Y. (2015). Three-dimensional ordered ZnO/Cu_2_O nanoheterojunctions for efficient metal-oxide solar cells. ACS Appl. Mater. Interfaces.

[110] Dang V.Q., Trung T.Q., Kim D.I., Duy L.T., Hwang B.U., Lee D.W., Kim B.Y., Toan L.D., Lee N.E. (2015). Ultrahigh responsivity in graphene-ZnO nanorod hybrid uv photodetector. Small.

[111] Nasiri N., Bo R., Wang F., Fu L., Tricoli A. (2015). Ultraporous electron-depleted ZnO nanoparticle networks for highly sensitive portable visible-blind UV photodetectors. Adv Mater..

[112] Lu J., Xu C., Dai J., Li J., Wang Y., Lin Y., Li P. (2015). Improved UV photoresponse of ZnO nanorod arrays by resonant coupling with surface plasmons of Al nanoparticles. Nanoscale.

[113] Jin Z., Zhou Q., Chen Y., Mao P., Li H., Liu H., Wang J., Li Y. (2016). Graphdiyne: ZnO nanocomposites for high-performance UV photodetectors. Adv. Mater..

[114] Pawar A.U., Kim C.W., Kang M.J., Kang Y.S. (2016). Crystal facet engineering of ZnO photoanode for the higher water splitting efficiency with proton transferable nafion film. Nano Energy.

[115] Mishra Y.K., Modi G., Cretu V., Postica V., Lupan O., Reimer T., Paulowicz I., Hrkac V., Benecke W., Kienle L., Adelung R. (2015). Direct growth of freestanding ZnO tetrapod networks for multifunctional applications in photocatalysis, UV photodetection, and gas sensing. ACS Appl. Mater. Interfaces.

[116] Yin M., Liu S. (2015). Controlled ZnO hierarchical structure for improved gas sensing performance. Sens. Actuator B.

[117] Kim H.W., Na H.G., Kwon Y.J., Cho H.Y., Lee C. (2015). Decoration of Co nanoparticles on ZnO-branched SnO_2_ nanowires to enhance gas sensing. Sens. Actuator B.

[118] Sun Y., Sun Y., Zhang T., Chen G., Zhang F., Liu D., Cai W., Li Y., Yang X., Li C. (2016). Complete Au@ZnO core-shell nanoparticles with enhanced plasmonic absorption enabling significantly improved photocatalysis. Nanoscale.

[119] Lu M.Y., Tsai C.Y., Chen H.A., Liang Y.T., Chen K.P., Gradečak S., Gwo S., Chen L.J. (2016). Plasmonic enhancement of Au nanoparticle—embedded single-crystalline ZnO nanowire dye-sensitized solar cells. Nano Energy.

[120] Zhang C., Shao M., Ning F., Xu S., Li Z., Wei M., Evans D.G., Duan X. (2015). Au nanoparticles sensitized ZnO nanorod@ nanoplatelet core–shell arrays for enhanced photoelectrochemical water splitting. Nano Energy.

[121] Sun M., Xu Z., Yin M., Lin Q., Lu L., Xue X., Zhu X., Cui Y., Fan Z., Ding Y., Tian L., Wang H., Chen X., Li D. (2016). Broad-band three dimensional nanocave ZnO thin film photodetectors enhanced by Au surface plasmon resonance. Nanoscale.

[122] Linic S., Christopher P., Ingram D.B. (2011). Plasmonic-metal nanostructures for efficient conversion of solar to chemical energy. Nat. Mater..

[123] Zhang X., Chen Y.L., Liu R.S., Tsai D.P. (2013). Plamonic photocatalysis. Rep. Prog. Phys..

